# Diet‐MEF2 interactions shape lipid droplet diversification in muscle to influence *Drosophila* lifespan

**DOI:** 10.1111/acel.13172

**Published:** 2020-06-14

**Authors:** Xiao Zhao, Xiaotong Li, Xiangyu Shi, Jason Karpac

**Affiliations:** ^1^ Department of Molecular and Cellular Medicine Texas A&M University Health Science Center Bryan TX USA

**Keywords:** carbohydrate, cyclin E, dietary adaptation, *Drosophila*, glucose, high calorie diet, lactate, lifespan, lipid droplet, lipid metabolism, MEF2, muscle, survival

## Abstract

The number, size, and composition of lipid droplets can be influenced by dietary changes that shift energy substrate availability. This diversification of lipid droplets can promote metabolic flexibility and shape cellular stress responses in unique tissues with distinctive metabolic roles. Using *Drosophila,* we uncovered a role for myocyte enhancer factor 2 (MEF2) in modulating diet‐dependent lipid droplet diversification within adult striated muscle, impacting mortality rates. Muscle‐specific attenuation of MEF2, whose chronic activation maintains glucose and mitochondrial homeostasis, leads to the accumulation of large, cholesterol ester‐enriched intramuscular lipid droplets in response to high calorie, carbohydrate‐sufficient diets. The diet‐dependent accumulation of these lipid droplets also correlates with both enhanced stress protection in muscle and increases in organismal lifespan. Furthermore, MEF2 attenuation releases an antagonistic regulation of cell cycle gene expression programs, and up‐regulation of Cyclin E is required for diet‐ and MEF2‐dependent diversification of intramuscular lipid droplets. The integration of MEF2‐regulated gene expression networks with dietary responses thus plays a critical role in shaping muscle metabolism and function, further influencing organismal lifespan. Together, these results highlight a potential protective role for intramuscular lipid droplets during dietary adaptation.

## Graphical abstract

Lipid droplets are key components in cells that exert a multitude of relevant functions in diverse tissues. We exploited diet‐gene interactions that control the size and composition of lipid droplets in muscle tissue, and further show that intramuscular accumulation of these organelles correlates with beneficial outcomes that includes diminished mortality rates. Intramuscular lipid droplet accumulation is adjusted by the integration of MEF2‐regulated gene expression networks with dietary responses, further influencing muscle tissue health and organismal lifespan.

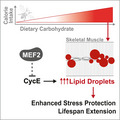

## 1 INTRODUCTION

Lipid droplets (LD) are ubiquitous cellular organelles that direct lipid storage across taxa (Guo, Cordes, Farese, & Walther, 2009; Thiam & Beller, 2017). As the major site of neutral lipid storage, primarily triglycerides, and sterol esters, these organelles provide energy substrates for mitochondrial beta oxidation. Thus, lipid droplets play a central and ancestral role in cellular and organismal energy homeostasis, as well as lipid metabolism (Welte, 2015).

Accordingly, LDs are fundamentally important in cells and tissues that govern lipid metabolism and energy homeostasis, such as adipose tissue, the intestine, the liver, and muscle. Unique or specific cellular functions also guide the number, size, and composition of lipid droplets within these metabolic tissues (Thiam & Beller, 2017). For example, in adipocytes, extremely large lipid droplets are massively enriched in triglycerides in order to provide fatty acids for systemic energy substrate usage. LDs in adipose thus act as stored macronutrient reservoirs for other tissues to balance energy homeostasis in response to diurnal changes in feeding or activity, as well as during nutrient deprivation (Baskin, Winders, & Olson, 2015; Zhao & Karpac, 2017). Conversely, lipid droplets in striated muscle, primarily an energy usage tissue are smaller with more varied lipid compositions (Amati et al., 2011; Covington et al., 2017; Schrauwen‐Hinderling, Hesselink, Schrauwen, & Kooi, 2006; Smith, Soeters, Wüst, & Houtkooper, 2018). These intramuscular LDs are usually embedded within dense mitochondrial networks, likely to facilitate efficient shuttling of fatty acids into mitochondria in order to compensate for elevated energy demands (Covington et al., 2017; Schrauwen‐Hinderling et al., 2006). The diversification of LD numbers, size, and composition is thus heavily influenced by specific cellular functions and highlights the dynamism and adaptability of these organelles (Goodpaster & Sparks, 2017).

Intramuscular lipid droplet diversification is further shaped by diet and nutrient availability. Dietary adaptation within tissues is critical to balance energy homeostasis, promotes cellular stress responses, and maintains organismal viability in both energy‐replete and depleted states. In striated muscle, LD accumulation in response to dietary adaption has been increasingly linked to advantageous outputs, such as enhanced metabolic flexibility and maintenance of cellular and systemic metabolic function (Bosma et al., 2012; Daemen, van Polanen, & Hesselink, 2018; Goodpaster & Sparks, 2017; Muoio, 2012; Smith et al., 2018; Wang et al., 2011; Watt, 2009). Thus, the diversification of intramuscular lipid droplets influenced by unique dietary challenges may reflect a subsequent diversification in lipid droplet function. To this end, accumulating evidence suggests that the biological roles of these organelles extend far beyond the control of energy homeostasis as macronutrient reservoirs (Welte, 2015; Welte & Gould, 2017). LDs have emerged as important nodes for various stress responses, including autophagy, innate immunity, anti‐oxidant mechanisms, and proteostasis (through controlling endoplasmic reticulum [ER] stress and the unfolded protein response [UPR], reviewed in (Henne, Reese, & Goodman, 2018)and (Anand et al., 2012; Bailey et al., 2015)). Since intramuscular LDs can potentially shape both metabolic flexibility and cellular stress responses, it is critical to explore the functional role of muscle lipid droplet diversification in response to dietary adaptation.

Genetically, tractable insect models provide unique advantages to explore the diversification of intramuscular lipid droplets in vivo. Flying insects have evolved specialized and high‐output flight muscles that require abundant energy substrates. To this end, these animals must readily adapt to changes in diet, either in macronutrient availability or calorie intake, to maintain muscle form and function. Here, we leverage in vivo high‐throughput LD screening in the fruit fly *Drosophila melanogaster* in order to uncover a diet‐dependent and myocyte enhancer factor 2 (MEF2)‐mediated diversification of intramuscular lipid droplets that correlates with enhanced lifespan.

## 2 RESULTS

### 2.1 MEF2 restrains intramuscular lipid droplet accumulation in response to high calorie diets

**FIGURE 1:**
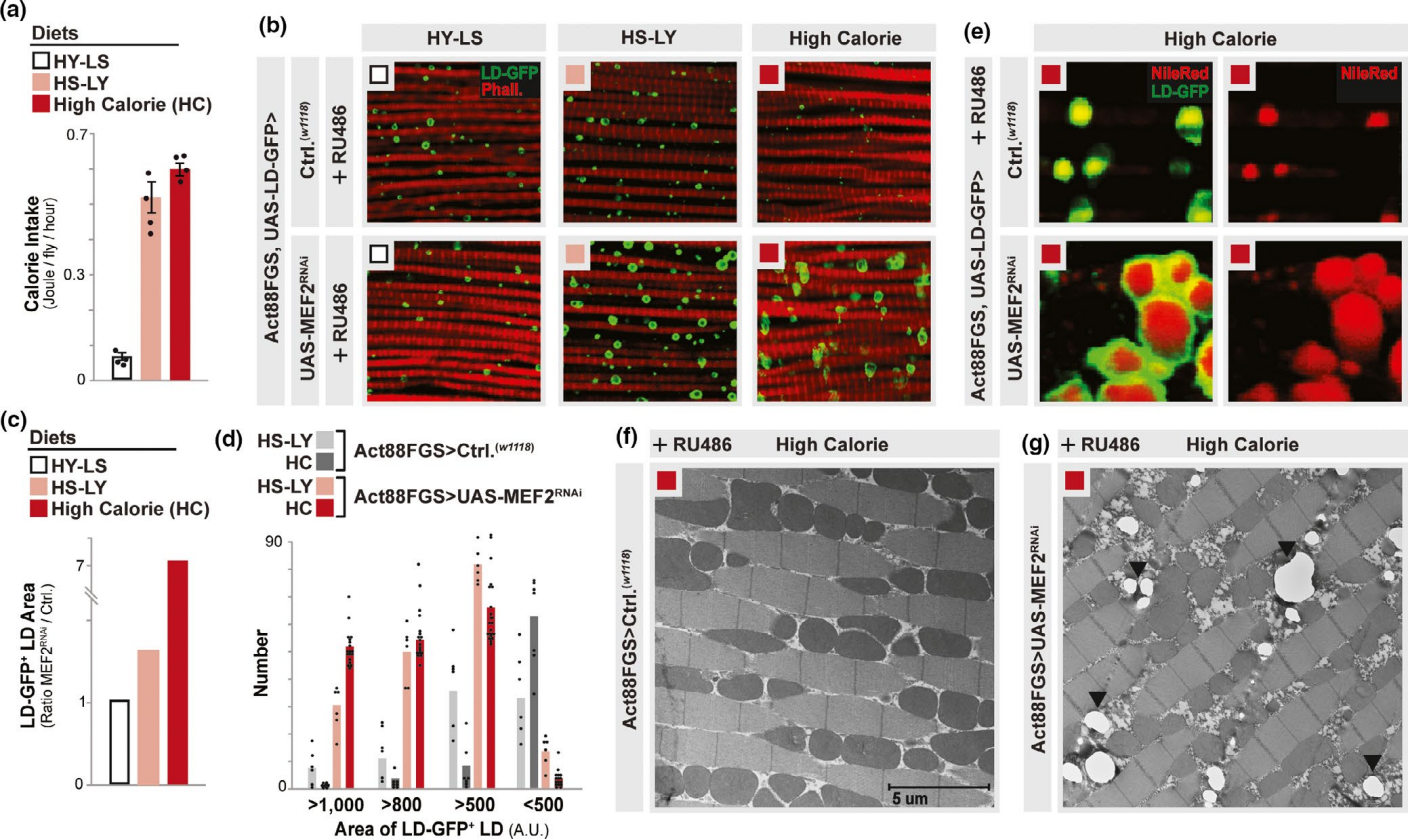
Diet‐MEF2 interactions shape intramuscular lipid droplet diversification. (a) Caloric intake related to indicated diets (joule/fly/hour). *n* = 4 samples. HS‐LY and HC diets display statistically significant increases in calorie intake compared to a HY‐LS diet. (b‐e) Diet‐ and MEF2‐dependent changes in lipid droplet accumulation. Genotypes *w^1118^*; Act88FGS, UAS‐LD‐GFP,/+ (Act88FGS, UAS‐LD‐GFP>*w^1118^*) or *w^1118^;* Act88FGS, UAS‐LD‐GFP/+; UAS‐MEF2 RNAi/+ (Act88FGS, UAS‐LD‐GFP>UAS‐MEF2 RNAi); all flies fed RU486. (b) Fluorescence imaging and staining to detect lipid droplets (LD) in dissected dorsal longitudinal thoracic (IFM) muscle from indicated diets; LD (LD‐GFP, green) and F‐actin filaments (Phalloidin, Red). (c) Quantification of LD size/area (LD‐GFP‐positive lipid droplets) from indicated diets (plotted as ratio of MEF2 RNAi size/Ctrl. [*w^1118^*] size). *n* = 480–1,250 LDs from 6–18 independent dissected muscle segments. (d) Quantification of LD size/area (LD‐GFP‐positive lipid droplets) from indicated diets (plotted as number of individual droplets of various sizes [A.U., arbitrary units >1,000, >800, >500, or <500]). *n* = 480–1,250 LDs from 6–18 independent dissected muscle segments. (e) Staining (40×) of LDs in dissected dorsal longitudinal thoracic (IFM) muscle (high calorie diet); LD (LD‐GFP, green) and neutral lipids (Nile Red, Red). (f‐g) Thoracic muscle ultrastructure visualized by electron microscopy; from (f) *w^1118^;* Act88FGS/+ (Act88FGS>*w^1118^*) or (g) *w^1118^; *Act88FGS/+; UAS‐MEF2 RNAi/+ (Act88FGS>UAS‐MEF2 RNAi); all flies fed RU486. Examples of LDs marked by black arrows, scale bars shown. Images are representative

Similar to numerous vertebrate somatic muscle types, *Drosophila* indirect flight muscle (thoracic IFM) is a cross‐striated fibrillar muscle, containing longitudinal and ventral segments, with similar contraction/relaxation regulatory mechanisms (Iwamoto, 2011; Swank, 2012). The indirect flight muscle is dotted with small lipid droplets in between myofibrils that can be efficiently labeled and visualized with a lipid‐droplet‐binding domain‐GFP fusion protein (UAS‐LD‐GFP) coupled with thoracic muscle‐specific transgenic expression drivers (Act88FGal4 and Act88FGeneSwitch[GS], Mlih, Khericha, Birdwell, West, & Karpac, 2018; Zhao & Karpac, 2017)). Using these genetic tools, we tested whether macronutrient composition of various energy‐replete diets could influence intramuscular LD diversification in *Drosophila* (mated female flies [3–4 days posteclosion] exposed to diets for 10 days). Diets include: (I) a high yeast–low sugar diet (HY‐LS; excessive in protein, which leads to a lower rate of calorie intake), (II) a high sugar–low yeast diet (HS‐LY; excessive in simple sugars [sucrose], which leads to a higher rate of calorie intake), and (III) a high calorie diet (slightly more balanced in macronutrients, but with sufficient carbohydrate [both simple and complex] to increase rates of calorie intake; Figure 1a and Figure S1a). Calorie intake was calculated based on feeding rate and the nutritional parameters of each diet. While all of these diets should provide excessive calories based on macronutrient composition and availability, distinct nutrient‐dependent changes in animal feeding alters the overall caloric intake (Figure 1a and (Skorupa, Dervisefendic, Zwiener, & Pletcher, 2008; Prasad & Hens, 2018)). However, measuring LD‐GFP+ lipid droplet area in IFM dorso‐longitudinal muscles (DLM), we found that HY‐LS and HS‐LY diets just nominally increased LD size compared to a high calorie diet (using both Act88FG4 and RU486‐dependent Act88FGS thoracic muscle drivers, Figure 1b and Figure S1b,c). Intermyofibrillar lipid droplets are intermixed with mitochondrial networks likely to maximize lipid energy substrate usage (Schrauwen‐Hinderling et al., 2006; Zhao & Karpac, 2017), so we next explored genetic modifications (linked to cellular energy homeostasis) that could potentially influence diet‐dependent intramuscular LD diversification. To this end, we uncovered a role for *Drosophila* MEF2 in modulating muscle LD size and composition.

MEF2 is an evolutionarily conserved transcription factor that directs various cellular functions including muscle cell differentiation during embryogenesis, as well as developmental myogenesis, across taxa (Bryantsev, Baker, Lovato, Jaramillo, & Cripps, 2012; Potthoff & Olson, 2007; Soler, Han, & Taylor, 2012). *Drosophila* MEF2 activity is maintained in adult thoracic IFM despite the completion of myogenesis, but MEF2 has been shown to control various aspects of energy homeostasis (i.e., lipid metabolism and aerobic respiration) in diverse tissues (Azzouzi et al., 2010; Bryantsev et al., 2012; Clark et al., 2013; Czubryt & Olson, 2004). MEF2 transcriptional activation function has also been shown to regulate mitochondrial function (Czubryt, McAnally, Fishman, & Olson, 2003; Michael et al., 2001). Thus, we assayed diet‐dependent intramuscular LD diversification in *Drosophila* with attenuated MEF2 activity. Inhibiting MEF2 specifically in adult thoracic muscle (Act88FGS, UAS‐LD‐GFP>UAS‐MEF2^RNAi ^+RU486; RNAi induced after adult myogenesis is complete) promotes the accumulation of large intramuscular lipid droplets in response to HS‐LY and high calorie diets, but not a HY‐LS diet (compared to controls; Act88FGS, UAS‐LD‐GFP>*w^1118^* +RU486, Figure 1b‐d; additional experimental analysis [Act88FGS>UAS‐MEF2^RNAi ^+/−RU486] is provided in Figure S2a). This is marked during high calorie diet feeding, where, on average, lipid droplet area is increased nearly 7‐fold (Figure 1c and Figure S2b), and we subsequently confirmed that these large LD‐GFP+ lipid droplets store neutral lipids (Figure 1e). Similar results were found using an independent MEF2^RNAi^ transgene (Figure S2c,d). Additionally, attenuating MEF2 with the Act88FGal4 driver promoted massive increases in LD size as well. This genetic manipulation, however, also disrupted myogenesis since the driver is active during the last stages of *Drosophila* muscle development (Figure S2e), highlighting the requirement to use adult‐inducible drivers to explore these diet‐dependent phenotypes. Finally, utilizing electron microscopy, we analyzed muscle ultrastructure to confirm the presence of large, intermyofibrillar LDs when MEF2 function is attenuated during high calorie diet feeding (Act88FGS>UAS‐MEF2^RNAi ^+RU486 compared to Act88FGS>*w^1118^* +RU486 controls, absent the LD‐GFP marker, Figure 1f,g). Taken together, these data show that *Drosophila* MEF2 activity in adult muscle (postmyogenesis) restrains the accumulation of large lipid droplets in response to carbohydrate‐sufficient high calorie diets.

**FIGURE 2:**
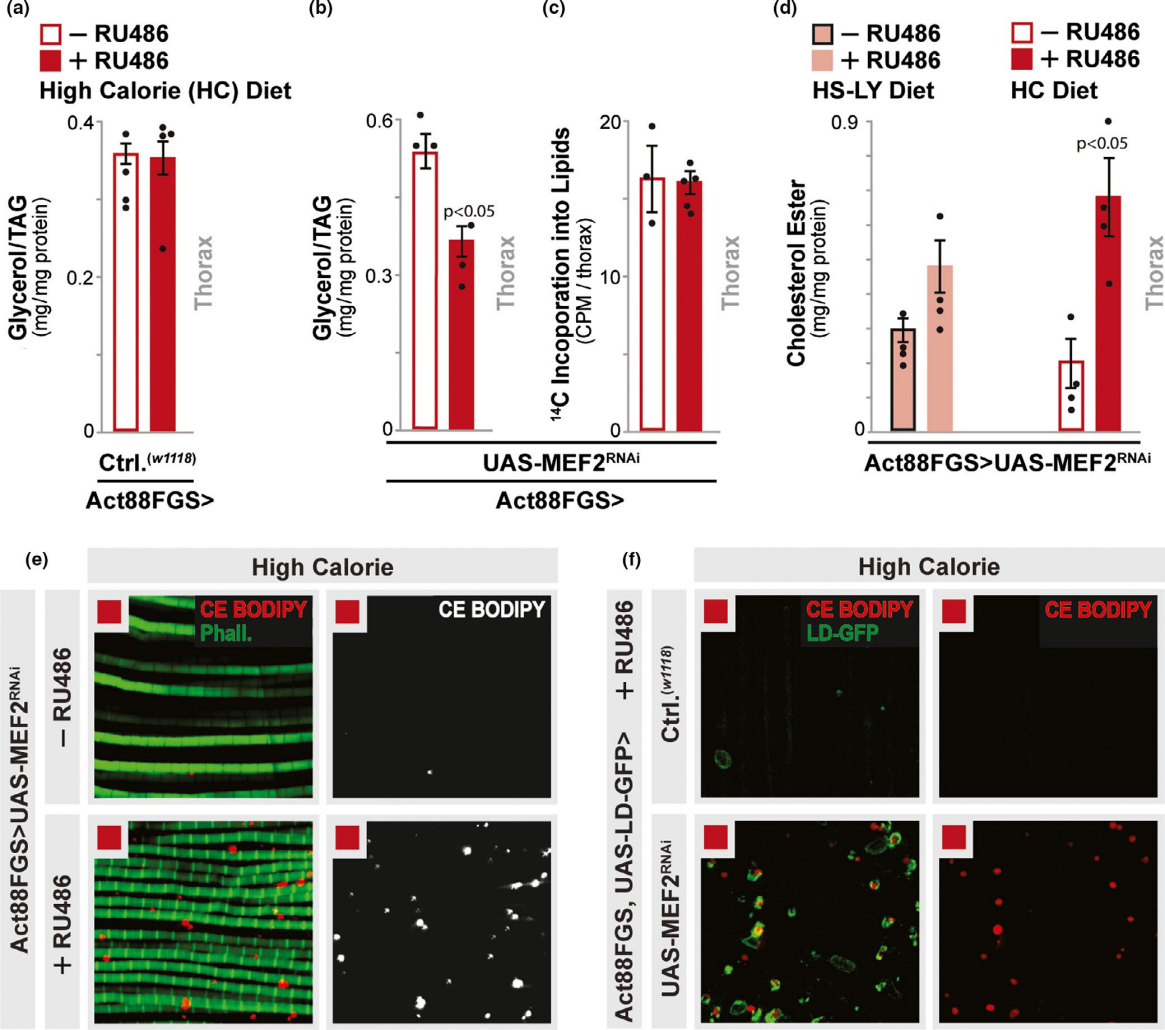
Diet‐MEF2 interactions shape intramuscular lipid content. (a) Triglyceride (TAG) content from dissected thoraces/muscle. *w^1118^;* Act88FGS/+ (Act88FGS>*w^1118^*) flies, +/−RU486 (high calorie diet). *n* = 4 samples. (b) TAG content from dissected thoraces/muscle. *w^1118^;* Act88FGS/+; UAS‐MEF2 RNAi/+ (Act88FGS>UAS‐MEF2 RNAi) flies, +/−RU486 (high calorie diet). *n* = 3 samples. (c) Quantification of de novo lipid synthesis via incorporation of diet‐fed (4 hr.) ^14^C‐labeled glucose into total lipids from dissected thoraces/muscle. *w^1118^;* Act88FGS/+; UAS‐MEF2 RNAi/+ (Act88FGS>UAS‐MEF2 RNAi) flies, +/−RU486 (high calorie diet). *n* = 3–5 samples. (d) Cholesterol ester content from dissected thoraces/muscle from indicated diets. *w^1118^;* Act88FGS/+; UAS‐MEF2 RNAi/+ (Act88FGS>UAS‐MEF2 RNAi) flies, +/−RU486. *n* = 4 samples. (e,f) Fluorescent imaging and staining of LDs in isolated/dissected dorsal longitudinal thoracic (IFM) muscle treated ex vivo with CholEsteryl (CE) BODIPY C11; (e) *w^1118^;* Act88FGS/+; UAS‐MEF2 RNAi/+ (Act88FGS>UAS‐MEF2 RNAi) flies; +/−RU486; F‐actin filaments (Phalloidin, Green) and CE BODIPY (Red/White). Red squares; HC diet. (f) *w^1118^;* Act88FGS, UAS‐LD‐GFP/+ (Act88FGS, UAS‐LD‐GFP>*w^1118^*) and *w^1118^;* Act88FGS, UAS‐LD‐GFP/+; UAS‐MEF2 RNAi/+ (Act88FGS, UAS‐LD‐GFP>UAS‐MEF2 RNAi); all flies fed RU486; LD (LD‐GFP, green) and CE BODIPY (Red). All flies fed a high calorie diet. Bars represent mean ± *SE*. All flies were females. Red squares; HC diet

The accumulation LDs within striated muscle is often associated with elevated muscle triglyceride content (Watt, 2009). Furthermore, most animals, and especially insects, rapidly convert excess carbohydrates into stored lipids through de novo synthesis in order to drive long‐term energy storage and promote dietary sugar tolerance (Havula et al., 2013; Zhao & Karpac, 2017). Contrastingly, we found that MEF2‐ and high calorie diet‐dependent LD accumulation correlates with decreased thoracic muscle triglyceride content in *Drosophila* (dissected thoraces enriched in IFM from Act88FGS>UAS‐MEF2^RNAi^ [+/−RU486] flies, Figure 2b). Additional controls (including RU486 dosing effects) are provided in Figure 2a and Figure S2f,g. There is also no difference in thoracic muscle de novo lipid synthesis (measured by incorporation of radiolabeled glucose (^14^C‐glucose) into total lipids extracted from dissected thoraces) in similar genetic backgrounds (Figure 2c). Besides triglycerides, lipid droplets can also act as reservoir for cholesterol (stored as esterified cholesterol), which is essential for maintaining cellular and systemic cholesterol homeostasis (Guo et al., 2009; Thiam & Beller, 2017; Walther, Chung, & Farese, 2016; Welte, 2015). To this end, we uncovered that MEF‐2 attenuation leads to cholesterol ester accumulation in thoracic muscle in response to HS‐LY and high calorie diets (Figure 2d), the strength of which correlates with the increasing size of LDs (Figures 1b and 2d), suggesting that many of these LDs contain cholesterol ester. To validate these biochemical assays, we utilized isolated longitudinal IFM muscle segments (ex vivo) coupled with a fluorescent cholesterol ester lipophilic dye (CholEsteryl [CE] BODIPY, (Hsieh et al., 2012)) and imaging. We found that muscle‐specific inhibition of MEF2 promotes strong increases in CE BODIPY‐positive intermyofibrillar LDs (Figure 2e, flies fed a high calorie diet), and most (but not all) diet‐and MEF2‐augmented LD‐GFP+ lipid droplets are enriched with cholesterol ester (Figure 2f). *Drosophila* MEF2 function thus influences diet‐dependent intramuscular lipid droplet diversification (both size and composition) linked to changes in calorie intake and/or macronutrient availability.

### 2.2 MEF2‐mediated and diet‐dependent accumulation of intramuscular lipid droplets correlates with enhanced organismal lifespan

**FIGURE 3:**
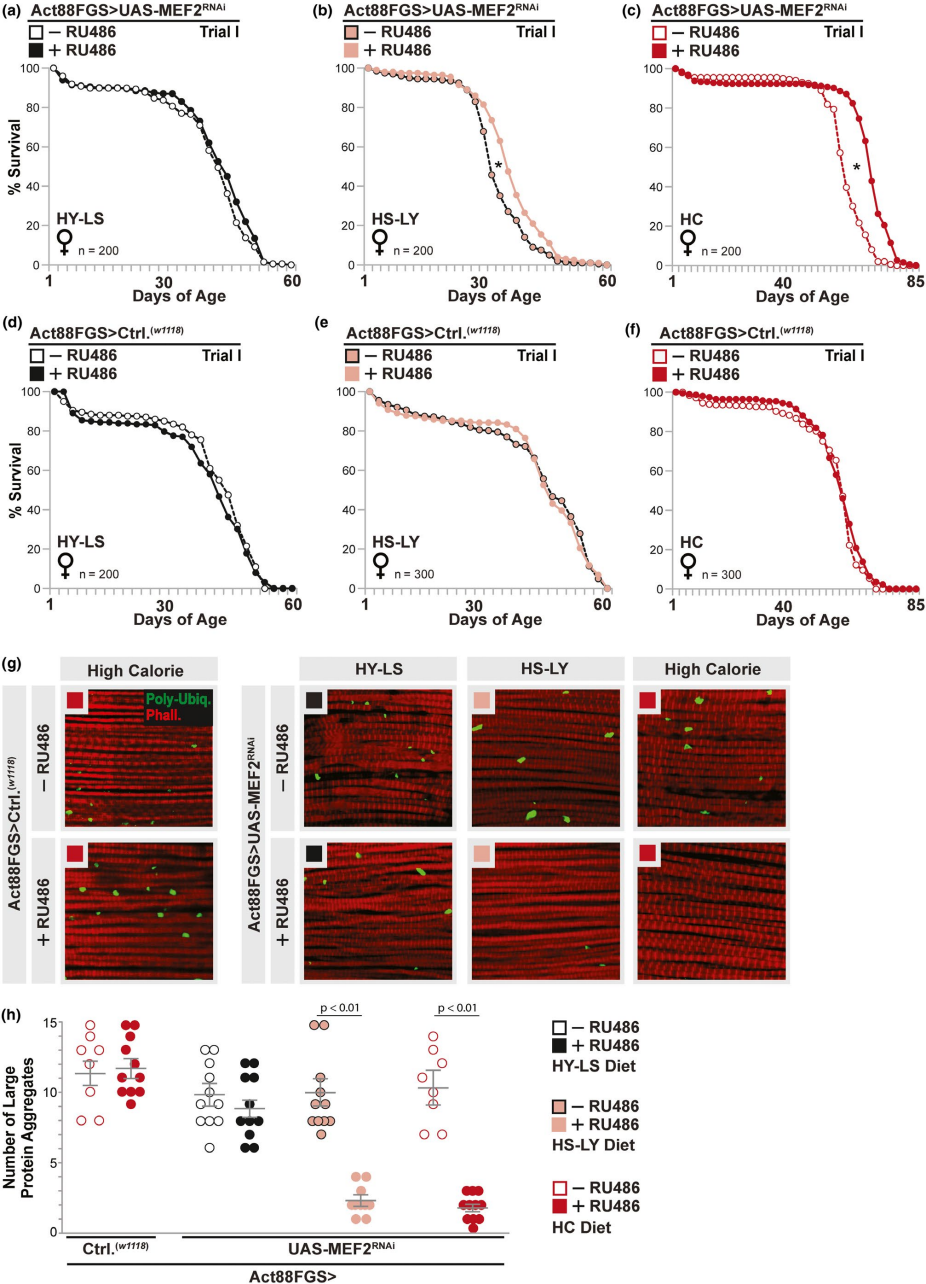
Intramuscular lipid droplet diversification correlates with stress protection and enhanced lifespan. (a‐f) Lifespan curves (lifespan, independent trial); *w^1118^;*Act88FGS/+;UAS‐MEF2 RNAi/+(Act88FGS>UAS‐MEF2 RNAi) female flies (+/−RU486) fed a (a) HY‐LS diet, (b) HS‐LY diet, or (c) high calorie (HC) diet; *w^1118^;* Act88FGS/+ (Act88FGS>*w^1118^*) female flies (+/−RU486) fed a (d) HY‐LS diet, (e) HS‐LY diet, or (f) HC diet. *n* = 200–300 flies; *p value <.01 (mean lifespan). (g) Immunostaining to detect poly‐ubiquitin protein (aggregates, Poly‐Ubiq.) in dissected dorsal longitudinal thoracic muscle from indicated diets; anti–poly‐ubiquitin (green), F‐actin filaments (Phalloidin, Red), and nuclei (DAPI, blue). Genotypes (+/−RU486) described above. Black squares; HY‐LS diet, Pink squares; HS‐LY diet, Red squares; HC diet. (h) Quantification of large protein aggregates (Poly‐Ubiq.‐positive) from indicated diets and genotypes. Circles represent independent (*n* = 8–11 dissected dorsal longitudinal thoracic (IFM) muscle segments. All flies were females

We next wanted to explore the functional consequences of these diet‐dependent changes in muscle lipid droplet size and composition. The utilization of *Drosophila* to tissue‐specifically and diet‐dependently modify intramuscular LD diversification allows for a more direct correlation between lipid droplet accumulation, tissue homeostasis, and organismal health. We uncovered that attenuating MEF2 function in thoracic muscle enhanced lifespan of *Drosophila* chronically fed a HS‐LY or high calorie diet, but not a HY‐LS diet (Act88FGS>UAS‐MEF2^RNAi^ +/−RU486, Figure 3a‐c and Figure S3a‐c). Mean lifespan is increased, while age‐specific mortality is decreased (Figure 3a‐c and Figure S3a‐g; additional RU486 controls are provided in Figure 3d‐f, and independent population/trial statistics [females and males] are provided in Table S1). Enhanced lifespan thus correlates with diet‐dependent intramuscular LD accumulation (Figure 1b‐d), not MEF2 inhibition. Furthermore, the extent of mean lifespan extension correlates with the extent of LD accumulation in muscle (i.e., mean lifespan extension is most pronounced in *Drosophila* fed a high calorie diet, where MEF2‐mediated intramuscular LD size is most increased compared to controls, Figure 3c and Figure 1c). Similar results were found using an independent MEF2^RNAi^ transgene (Figure S3h‐i). While dietary macronutrient imbalances can significantly reduce lifespan (HY‐LS and HS‐LY), LD diversification is still positively associated with lifespan extension in flies fed an imbalanced HS‐LY diet (Figure 3b).

We also found evidence that diet‐dependent accumulation of LDs in muscle autonomously enhances stress protection. Maintaining muscle proteostasis (marked by stress‐dependent changes in intramuscular protein aggregates) can often improve organismal survival rates, especially in *Drosophila* (Bai, Kang, Hernandez, & Tatar, 2013; Demontis & Perrimon, 2010). LD diversification in longitudinal IFM, promoted by MEF2 attenuation and carbohydrate‐sufficient high calorie diets, also correlates with decreases in intramuscular protein aggregates (Act88FGS>UAS‐MEF2^RNAi^ +/−RU486, and additional Act88FGS>*w^1118^* +/−RU486 controls; Figure 3g,h). Again, enhanced muscle proteostasis correlates with LD accumulation, as there is no MEF2‐dependent effect on protein aggregates in response to a HY‐LS diet (Figure 3g,h).

In summary, these results suggest that lipid droplet diversification in muscle, related to changes in distinctive dietary macronutrients and/or calorie intake, may play an overall protective role in maintaining tissue homeostasis and subsequently enhance organismal lifespan.

### 2.3 MEF2‐regulated transcriptional networks govern intramuscular lipid droplet diversification in response to availability of specific dietary macronutrients

**FIGURE 4:**
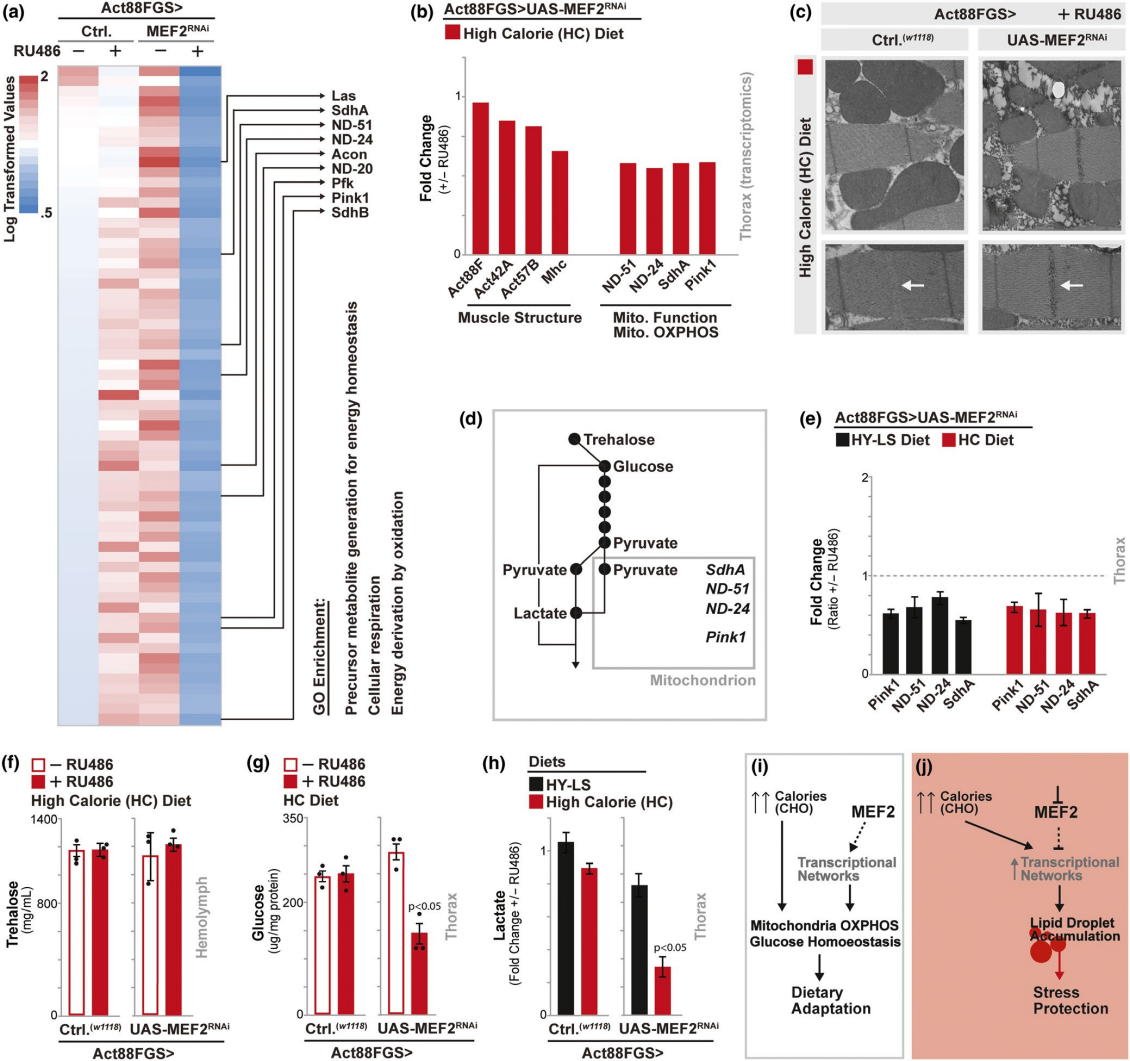
MEF2 function in muscle governs gene expression networks required to maintain glucose homeostasis in response to dietary adaptation (a) Heat map of gene expression levels (Log_2_ transformed values) from RNA‐seq. transcriptomics analysis (dissected thoraces/muscle) from *w^1118^;* Act88FGS/+ (Act88FGS>*w^1118^*) and *w^1118^;* Act88FGS/+; UAS‐MEF2 RNAi/+ (Act88FGS>UAS‐MEF2 RNAi) flies (+/−RU486, high calorie diet); some example genes provided. Map highlights genes down‐regulated in MEF2 RNAi flies (fold change <=0.65 MEF2 RNAi +RU486/‐RU486 without down‐regulation in controls). Down‐regulated genes were analyzed for Gene Ontology (GO) terms (FlyMine, enrichment *p* value <.001). (b) Fold change (Log_2_ transformed transcriptomic values, +RU486/‐RU486) of select genes involved in muscle structure or mitochondria function/oxidative phosphorylation (genotypes and diets described above). (c) Thoracic muscle ultrastructure visualized by electron microscopy; from *w^1118^;* Act88FGS/+ (Act88FGS>*w^1118^*) and *w^1118^;* Act88FGS/+; UAS‐MEF2 RNAi/+ (Act88FGS>UAS‐MEF2 RNAi); all flies fed RU486. Focusing on: (I) mitochondria (top panels), and (II) sarcomeres (bottom panels). White arrow designates ultrastructural changes to H‐zone (MEF2 RNAi). (d) Schematic depicting the breakdown of trehalose to glucose, pyruvate, and lactate in the control of energy homeostasis (including the requirements of mitochondria and nuclear‐encoded mitochondrial genes SdhA, ND‐51, ND‐24, and Pink1). (e) *Drosophila* *pink1*, *ND‐51*, *ND‐24*, and *sdhA* transcription (measured by qRT‐PCR from dissected thoraces/muscle, plotted as fold change [ratio +RU486/‐RU486]). Genotype *w^1118^;* Act88FGS/+; UAS‐MEF2 RNAi/+ (Act88FGS>UAS‐MEF2 RNAi); from indicated diets. *n* = 4 samples, all changes are statistically significant. (f) Trehalose levels from isolated hemolymph. *w^1118^;* Act88FGS/+ (Act88FGS>*w^1118^*) and *w^1118^;* Act88FGS/+; UAS‐MEF2 RNAi/+ (Act88FGS>UAS‐MEF2 RNAi) flies, +/−RU486 (high calorie diet). *n* = 3 samples. (g) Glucose levels from dissected thoraces/muscle. Genotypes described above, *n* = 3 samples. (h) Lactate levels from dissected thoraces/muscle. Genotypes described above, from indicated diets. Plotted as fold change (ratio +RU486/‐RU486). *n* = 3–4 samples. (i) Model depicting MEF2 (muscle) transcriptional activation function control of mitochondria function and glucose homeostasis in response to dietary CHO (carbohydrates). (j) Model depicting putative MEF2‐(muscle) dependent regulation of lipid droplet diversification. Bars represent mean ± *SE*. All flies were females

Our data highlight that MEF2 function restrains LD accumulation in indirect flight muscle when *Drosophila* are exposed to diets that increase caloric intake, driven by sufficient levels of dietary carbohydrates. Unique tissues with diverse functions can differentially utilize dietary macronutrients as energy substrates, and sugars act as essential (and efficient) carbon sources for fueling flight muscles in both invertebrates and hovering vertebrates (Jenni‐Eiermann, 2017; Sacktor & Childress, 1967; Suarez et al., 2005; Welch & Chen, 2014). Thus, we next wanted to explore MEF2 dependent muscle transcriptional networks, putatively influenced by carbohydrate availability, that could modulate lipid droplet diversification and potentially alter organismal lifespan. Utilizing transcriptomics (RNA‐seq.), we generated genome‐wide expression profiles from dissected thoraces of Act88FGS>UAS‐MEF2^RNAi^ (+/−RU486) flies fed a high calorie diet (compared to controls [Act88FGS>*w^1118^* +/−RU486]). Gene Ontology (GO) clustering analysis revealed that almost all significantly over‐represented GO terms associated with down‐regulated genes in response to MEF2 attenuation were related to oxidative phosphorylation (and thus mitochondrial) regulation of energy homeostasis (Figure 4a and Figure S4a). While protein‐coding genes involved in mitochondrial oxidative phosphorylation (OXPHOS) of energy substrates (such as ND‐51 and 24 [NADH dehydrogenase] and SdhA [succinate dehydrogenase, A]) and mitochondrial function (such as Pink1[PTEN‐induced putative kinase 1]) are down‐regulated, muscle structure genes are minimally changed (comparing fold change in Log_2_ transformed values between Act88FGS>UAS‐MEF2^RNAi^ [+/−RU486] and Act88FGS>*w^1118^* [+/−RU486], Figure 4b). This is consistent with previous reports describing that MEF2 transcriptional activation function is dispensable for maintenance of structural genes in the adult IFM (Bryantsev et al., 2012), highlighting additional functions for MEF2 postmyogenesis. These MEF2‐dependent changes in gene expression are supported by muscle ultrastructure, as intermyofibrillar mitochondria are less in number and smaller in size (Figure 4c and Figure 1f,g). Contrastingly, there is little evidence of myopathy in Act88FGS>UAS‐MEF2^RNAi^ (+RU486) flies, apart from protein accumulation in the H‐zone, as sarcomere length, sarcomere width, and Z‐line architecture all appear normal (Figure 4c and Figure 1f,g).

The dense intermyofibrillar mitochondria networks also support efficient carbohydrate usage to generate energy for insect flight and motility. Briefly, the circulating disaccharide trehalose is converted to glucose in muscle cells. Through glycolysis, glucose is further converted to pyruvate for use in the citric acid cycle, supporting cellular respiration and energy production via oxidation (Figure 4d) and (Matsuda, Yamada, Yoshida, & Nishimura, 2014; Mattila & Hietakangas, 2017; Yoshida, Matsuda, Kubo, & Nishimura, 2016). Dysfunctional mitochondria or insufficient oxygen levels can drive mitochondria‐independent generation of energy (inefficiently) through the reduction of pyruvate to lactate (Figure 4d). We found that MEF2 is required to maintain mitochondrial OXPHOS genes in response to both high calorie (carbohydrate sufficient) and low calorie (carbohydrate deficient) diets (measured by qRT‐PCR, Figure 4e), without influencing systemic physiology or feeding behavior (Figure S5a‐f). However, these changes in mitochondria function correlate with a disruption of autonomous glucose homeostasis only in response to high calorie diets. Despite normal circulating trehalose levels (hemolymph, Figure 4f), muscle‐specific MEF2 attenuation during high calorie diet feeding leads to significant decreases in thoracic muscle glucose and lactate levels (from dissected thoraces, Figure 4g,h). Lactate, in particular, is normally maintained in muscle, and robust decreases imply a disruption in glucose/energy homeostasis brought about by mitochondrial dysfunction (Smith et al., 2018). Lactate levels are not changed in Act88FGS>UAS‐MEF2^RNAi^ (+RU486) flies in response to a HY‐LS diet (Figure 4h), suggesting that MEF2‐dependent control of mitochondrial function is required to promote energy homeostasis (production) when carbohydrates are readily available in the diet (Barry & Thummel, 2016; Yoon, Hwang, Lee, & Chung, 2019). To this end, MEF2 attenuation impacts fly motility only in response to high calorie or HS‐LY diets (Figure S5g‐i), highlighting a trade‐off between carbohydrate‐driven muscle function/energy metabolism and lipid droplet diversification, both modulated by MEF2 activity (Figure S5j).

In summary, MEF2 transcriptional activation function is required to maintain mitochondrial OXPHOS gene expression networks, and promote glucose homeostasis and muscle function, in response to carbohydrate availability (Figure 4i, supported by previous work (Czubryt et al., 2003; Michael et al., 2001)). However, directly inhibiting mitochondrial OXPHOS (ND‐24, UAS‐ND‐24^RNAi^; (Garcia, Khajeh, Coulanges, Chen, & Owusu‐Ansah, 2017)) or maintenance (Pink1, UAS‐Pink1^RNAi^; (Koehler, Perkins, Ellisman, & Jones, 2017)) genes in muscle (Act88FGS) does not significantly change intramuscular lipid droplet size or number (Figure S5k). Thus, we next wanted to determine if other MEF2‐dependent changes in gene expression were driving intramuscular lipid droplet diversification (Figure 4j). Since muscle mitochondrial dysfunction in *Drosophila* is often linked to decreased lifespan (Aparicio, Rana, & Walker, 2019; Rana et al., 2017; Walker & Benzer, 2004), we hypothesized that other MEF2‐dependent transcriptional networks, within adult striated muscle, were promoting LD accumulation that correlates with enhanced lifespan (Figure 4j).

**FIGURE 5:**
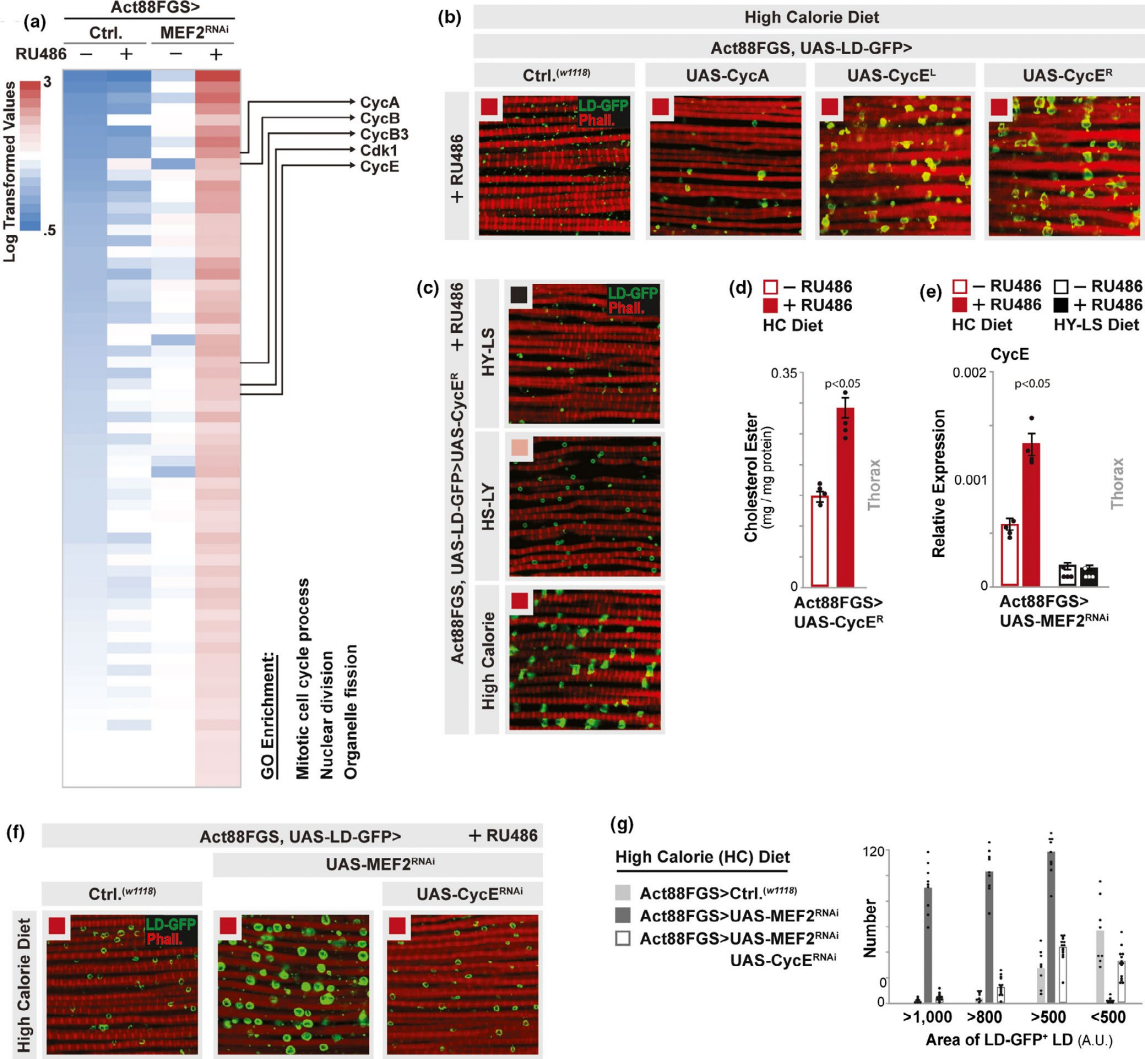
Diet‐ and MEF2‐dependent regulation of Cyclin E shapes intramuscular lipid droplet diversification. (a) Heat map of gene expression levels (Log_2_ transformed values) from RNA‐seq. transcriptomics analysis (dissected thoraces/muscle) from *w^1118^;*Act88FGS/+ (Act88FGS>*w^1118^*) and *w^1118^;*Act88FGS/+;UAS‐MEF2 RNAi/+ (Act88FGS>UAS‐MEF2 RNAi) flies (+/−RU486, high calorie diet); some example genes provided. Map highlights genes up‐regulated in MEF2 RNAi flies (fold change >=1.5 MEF2 RNAi +RU486/‐RU486 without up‐regulation in controls). Up‐regulated genes were analyzed for Gene Ontology (GO) terms (FlyMine, enrichment *p* value <.001). (b,c) Immunostaining to detect lipid droplets (LD) in dissected dorsal longitudinal thoracic (IFM) muscle; LD (LD‐GFP, green) and F‐actin filaments (Phalloidin, Red). Genotypes *w^1118^;* UAS‐LD‐GFP, Act88FGS/+ (Act88FGS, UAS‐LD‐GFP>*w^1118^*), or UAS‐CycA (*w^1118^;* Act88FGS, UAS‐LD‐GFP/UAS‐CycA), UAS‐CycE L (*w^1118^;* Act88FGS, UAS‐LD‐GFP/+; UAS‐CycE‐L/+), and UAS‐CycE R (*w^1118^;* Act88FGS, UAS‐LD‐GFP/UAS‐CycE R); all flies fed RU486 and (b) a high calorie diet or (c) a HY‐LS, HS‐LY, or high calorie diet. Black squares; HY‐LS diet. Pink squares; HS‐LY diet. Red squares; HC diet. (d) Cholesterol ester content from dissected thoraces/muscle (high calorie diet). *w^1118^;* Act88FGS, UAS‐CycE R/+ (Act88FGS>CycE R RNAi) flies, +/−RU486. *n* = 4 samples. (e) *Drosophila* *cycE* transcription (measured by qRT‐PCR from dissected thoraces/muscle, plotted as relative expression). Genotype *w^1118^;*Act88FGS/+;UAS‐MEF2 RNAi/+ (Act88FGS>UAS‐MEF2 RNAi) +/−RU486; from indicated diets. *n* = 4 samples. (f) Immunostaining to detect LDs in dissected dorsal longitudinal thoracic (IFM) muscle; LD (LD‐GFP, green) and F‐actin filaments (Phalloidin, Red). Genotypes described above, except for *w^1118^;* Act88FGS, UAS‐LD‐GFP/UAS‐CycE RNAi; UAS‐MEF2 RNAi/+ (Act88FGS, UAS‐LD‐GFP>UAS‐MEF2 RNAi, UAS‐CycE RNAi) all flies fed RU486. Red squares; HC diet. (g) Quantification of LD size/area (LD‐GFP‐positive lipid droplets) from genotypes (high calorie diet) described above (plotted as number of individual droplets of various sizes [A.U., arbitrary units >1,000, >800, >500, or <500]). *n* = 450–650 LDs from 6 independent dissected muscle segments. Bars represent mean ± *SE*. All flies were females

Again, using thoracic muscle transcriptomics, GO clustering analysis revealed that almost all significantly over‐represented GO terms associated with up‐regulated genes in response to MEF2 attenuation were related to the cell cycle process, including cyclins and cyclin‐dependent kinases (Figure 5a and Figure S4b). Cyclins have been shown to control the balance of cellular proliferation and differentiation during muscle regeneration or myogenesis (Giannattasio et al., 2018), and MEF2 isoforms have also been found to antagonize cell cycle gene expression profiles (Desjardins & Naya, 2017). Furthermore, cell cycle genes, dependent and independent of cell division, have emerged as critical regulators of cellular metabolism, including lipid droplet formation (Fajas, 2013). Thus, we tested whether over‐expression of two cyclin genes (Cyclin A [CycA] or E [CycE], previously shown to impact lipid droplet formation in *Drosophila* cell culture through unknown mechanisms (Guo et al., 2008)), could promote intramuscular LD accumulation. Over‐expressing CycA specifically in adult thoracic muscle (Act88FGS, UAS‐LD‐GFP>UAS‐CycA +RU486) had only minimal effect, while over‐expressing CycE (using two independent transgenic lines; UAS‐CycE^L^ and UAS‐CycE^R^, (Richardson, O'Keefe, Marty, & Saint, 1995)) promotes the accumulation of large intramuscular lipid droplets in response to high calorie diets (Figure 5b), similar to muscle‐specific MEF2 attenuation (Figure 1). CycE over‐expression enhanced lipid droplet diversification (size and composition [cholesterol ester]) in longitudinal IFM only during HS‐LY or high calorie diet feeding (Figure 5c,d) without impacting muscle structure/organization, glucose/lactate homeostasis, or motility (Figure S6a,b).

**FIGURE 6:**
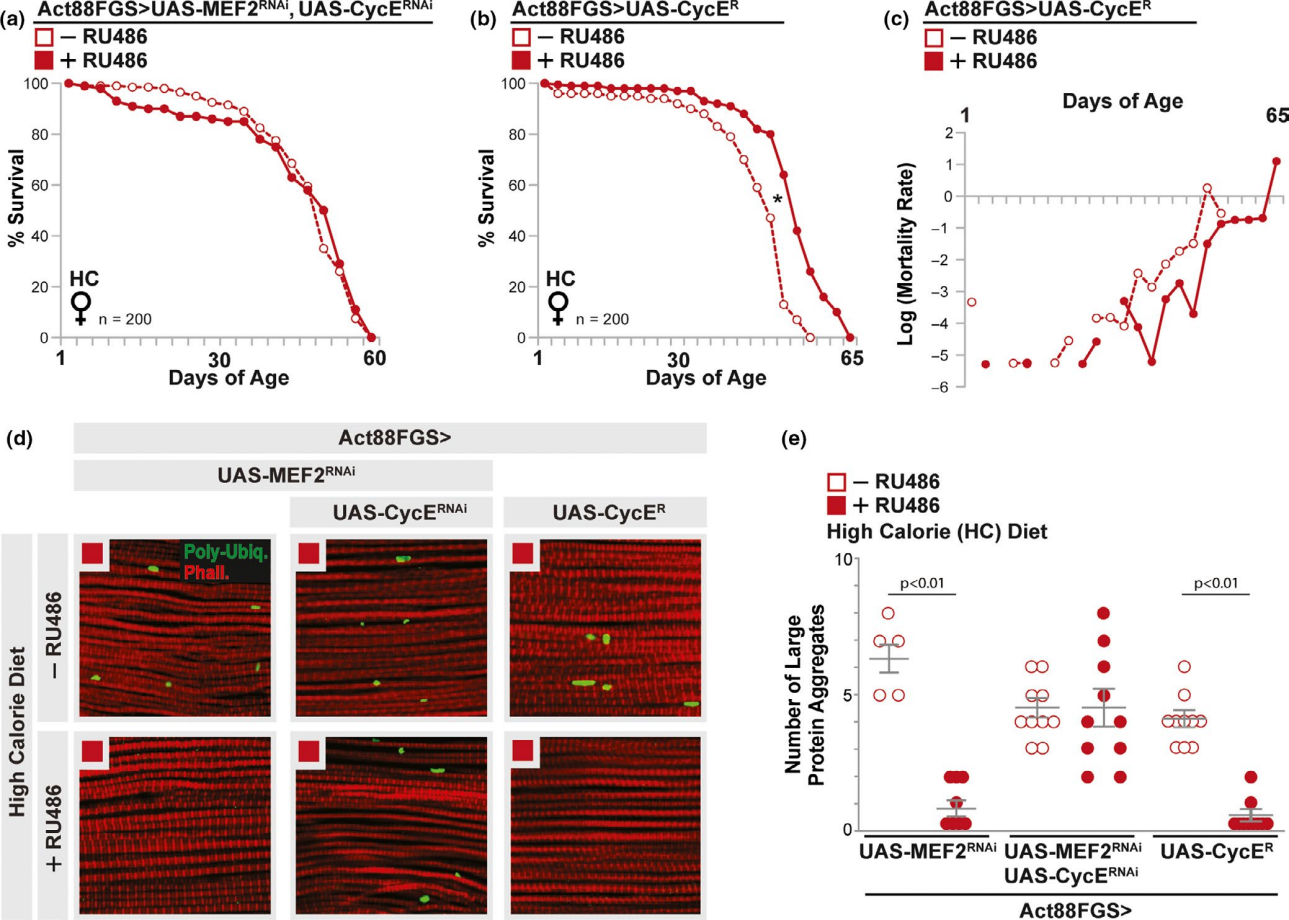
Diet‐ and MEF2‐dependent regulation of Cyclin E in muscle enhances stress protection and lifespan (a) Lifespan curve; *w^1118^;* Act88FGS, UAS‐LD‐GFP/UAS‐CycE RNAi; UAS‐MEF2 RNAi/+ (Act88FGS, UAS‐LD‐GFP>UAS‐MEF2 RNAi, UAS‐CycE RNAi) female flies (+/−RU486) fed a high calorie (HC) diet. *n* = 200 flies. (b) Lifespan curve; *w^1118^;* Act88FGS, UAS‐CycE R/+ (Act88FGS>CycE R RNAi) female flies (+/−RU486) fed a high calorie (HC) diet. *n* = 200 flies; *p value <.01 (mean lifespan). (c) Mortality plot; genotype described above (+/−RU486). (d) Immunostaining to detect poly‐ubiquitin protein (aggregates, Poly‐Ubiq.) in dissected dorsal longitudinal thoracic muscle from indicated diet; anti–poly‐ubiquitin (green), F‐actin filaments (Phalloidin, Red), and nuclei (DAPI, blue). Genotypes described above, female flies. Red squares ;HC diet. (e) Quantification of large protein aggregates (Poly‐Ubiq.‐positive) from indicated diet and genotypes. Circles represent independent (*n* = 5–10) dissected dorsal longitudinal thoracic (IFM) muscle segments

We next confirmed that MEF2 attenuation leads to up‐regulation of muscle CycE transcription in response to a high calorie diet (Figure 5e). However, thoracic muscle CycE expression levels are low in response to HY‐LS diets, and not controlled by MEF2 function (Figure 5e). Furthermore, genetically attenuating CycE in thoracic muscle (UAS‐CycE^RNAi^) could completely rescue intramuscular lipid droplet accumulation induced by MEF2 inhibition (Act88FGS>UAS‐MEF2^RNAi^ [+RU486]) in response to high calorie, carbohydrate‐sufficient diets (Figure 5f,g and Figure S6c,d). Thus, carbohydrate availability may modulate Cyclin E transcription, and diet‐MEF2 interactions that regulate gene expression networks are critical for shaping LD diversification in muscle that correlates with enhanced stress protection and organismal lifespan. To this end, CycE is required for high calorie‐ and MEF2‐dependent increases in lifespan (Figure 6a, Act88FGS>UAS‐MEF2^RNAi^, UAS‐CycE^RNAi^ +/−RU486), and CycE over‐expression alone enhances mean lifespan and decreases age‐specific mortality (Figure 6b,c, Act88FGS>UAS‐CycE^R^ +/−RU486). Furthermore, Cyclin E is both necessary (in the context of diet‐MEF2 interactions) and sufficient to modulate diet‐dependent changes in intramuscular protein aggregation (Figure 6d,e).

## 3 DISCUSSION

Taken together, these data highlight that diet‐MEF2 interactions in *Drosophila* can shape muscle form and function in response to the availability of dietary macronutrients. MEF2 function is required to promote mitochondria function, and thus maintain glucose/energy homeostasis when flies are fed high calorie (and carbohydrate sufficient) diets. Contrastingly, MEF2 function also restrains intramuscular lipid droplet accumulation in response to these same diets, and LD diversification positively correlates with enhanced organismal lifespan. This MEF2‐dependent trade‐off between maintenance of energy homeostasis and lipid droplet accumulation in insects might also inform on the complex relationship between lipid droplets and skeletal muscle in mammals. Obesogenic diets promote intramuscular lipid droplet accumulation, and the extent of changes in LD size and number often correlates with metabolic disease progression (Amati et al., 2011; Covington et al., 2017; Daemen et al., 2018). However, trained athletes also accumulate lipid droplets in skeletal muscle, suggested to promote metabolic flexibility during exercise (Amati et al., 2011; Li et al., 2019; Muoio, 2012). This paradox highlights the complexity of LD dynamics within muscle. The utilization of tissue‐specific insect genetic models to modulate muscle lipid droplet size and composition enables more direct correlations between lipid droplet diversification and organismal health. While not completely causal, our data suggest that diet‐dependent intramuscular LD accumulation may in fact be protective, promoting autonomous stress protection that can influence organismal lifespan.

Lipid droplets have functions beyond energy storage, and LDs (especially assembly of LDs) play a role in cellular stress responses (Henne et al., 2018; Welte, 2015). As lipid droplets bud from the endoplasmic reticulum (ER), major ER‐dependent stress response pathways (such as the unfolded protein response [UPR]) have been connected with LD production (Chen et al., 2017). Furthermore, lipid droplets function in protein quality control by influencing autophagosome production and acting as cytoplasmic chaperones (accumulating mis‐folded proteins) to prevent protein toxicity (Moldavski et al., 2015; Shpilka et al., 2015; Velázquez & Graef, 2016; Velázquez, Tatsuta, Ghillebert, Drescher, & Graef, 2016). Enhanced UPR stress responses and proteostasis in insect flight muscle are positively associated with lifespan (Owusu‐Ansah, Song, & Perrimon, 2013). The ability of these organelles to promote stress responses is likely aided by a diverse array of lipid‐droplet binding proteins that decorate LD surfaces (Anand et al., 2012; Welte, 2015). Thus, diet‐ and MEF2‐dependent accumulation of intramuscular LDs may impact tissue homeostasis and lifespan through promoting beneficial cellular stress responses to improve protein quality control. Furthermore, our data highlight that LD composition (i.e., cholesterol ester content) may also be an important determinant for LD‐specific roles in stress protection within striated muscle.

While lipid droplets are found in most cells, the biogenesis and/or growth of these organelles (potentially through unique mechanisms in unique tissues) is complex (reviewed in Walther et al., 2016; Wilfling, Haas, Walther, & Farese, 2014)). Our findings show that muscle‐specific, MEF2‐repressed transcriptional networks, in coordination with sufficient levels of dietary carbohydrates, can shift LD size and composition. MEF2 attenuation releases an antagonistic regulation of cell cycle gene expression programs, and up‐regulation of Cyclin E is required for diet‐ and MEF2‐dependent diversification of intramuscular lipid droplets. The adult *Drosophila* IFM is relatively postmitotic (Boukhatmi & Bray, 2018; Chaturvedi, Reichert, Gunage, & VijayRaghavan, 2017), suggesting that CycE governs LD diversification independent of cell cycle initiation. This regulation of LDs could be through re‐purposing cell cycle metabolic control mechanisms in postmitotic and metabolic cells, or through unique mechanisms divergent from cell division. It is also possible that CycE influences cholesterol homeostasis, within muscle, to impact lipid droplet formation and/or growth. While it remains unclear how Cyclin E dictates lipid droplet biology, numerous and varied experimental models support these findings, highlighting that cell cycle regulators (in proliferating and nonproliferating cells): (I) modulate autonomous and systemic metabolic processes, (II) are activated/regulated by carbohydrates such as glucose, and (III) trigger adaptive metabolic switches (Aguilar & Fajas, 2010; An et al., 2015; Fajas, 2013; Hanse et al., 2012; Heckmann, Zhang, Xie, & Liu, 2013; Long et al., 2012; Lopez‐Mejia, Castillo‐Armengol, Lagarrigue, & Fajas, 2018; Lucenay et al., 2016; Wu et al., 2019). This includes a potential role in lipid droplet diversification across taxa (Guo et al., 2008).

## 4 MATERIALS AND METHODS

### 4.1 *Drosophila* stocks and culture

The following strains were obtained from Bloomington Drosophila Stock Center: *w^1118^*; UAS‐MEF2^RNAi TRiP^ (TRiP; Bloomington 38247); UAS‐CycE.R (Bloomington 30725); UAS‐CycE.L (Bloomington 4781); UAS‐CycA (Bloomington 6633); UAS‐Luciferase^RNAi^ (Bloomington 31603); Act88FGal4 (Bloomington 38461); UAS‐Pink1^RNAi TRiP^ (Bloomington 31262), and UAS‐ND‐24^RNAi TRiP^ (Bloomington 51855). The following strains were obtained from Vienna Drosophila RNAi Center: UAS‐CycE^RNAi^ (GD47941) and UAS‐MEF2^RNAi^ (GD15549). The UAS‐LD‐GFP strain was kindly provided by M. Welte. Act88FGeneSwitch transgenic flies were generated and characterized in our lab (Thiam & Beller, 2017).

The high calorie (HC) diet (cornmeal‐based) was made with the following protocol: 10 g Agar/110 g Malt Extract/27.5 g Dry yeast/52 g Cornmeal/3.125 ml propionic acid/2 g Methyl 4‐Hydroxybenzoate/1.0 L water. The standard SY diet was made with the following protocol (as previously described in (Skorupa et al., 2008)): 10 g agar/100 g sucrose/100 g yeast/3.125 ml propionic acid/2 g Methyl 4‐Hydroxybenzoate/1.0 L water. The high sugar/low yeast diet was made with the following protocol: 10 g agar/400 g sucrose/25 g yeast/3.125 ml propionic acid/2 g Methyl 4‐Hydroxybenzoate/1.0 L water. The high yeast/low sugar diet was made with the following protocol: 10 g agar/25 g sucrose/400 g yeast/3.125 ml propionic acid/2 g Methyl 4‐Hydroxybenzoate/1.0 L water. Ingredients were combined, heated to 102°C for 1 hr, and cooled to 70°C before pouring. For RU486 food, RU486 (Sigma, M8046; dissolved in ethanol) or ethanol (‐RU486 control) was mixed with food, resulting in a 100 µM concentration of RU486 in the food, unless otherwise indicated.

A more detailed description of all materials and methods is provided in Supplemental Information.

## CONFLICT OF INTEREST

The authors declare no competing interests.

## AUTHOR CONTRIBUTIONS

X.Z. and X.L. designed and performed experiments, as well as wrote the manuscript. J.K. designed experiments and wrote the manuscript. X.S. performed lifespan experiments.

## Supporting information

Table S3

Supplementary Material

## Data Availability

The raw data that supports the lifespan and mortality findings of this study, as well as statistical analyses, are available in the supplementary information of this article. FASTQ data files representing unique RNA sequencing libraries were deposited in the NCBI Gene Expression Ominbus database (GSE147676). All other data that support the findings of this study are available from the corresponding author upon reasonable request.
