# Supplementary material for: Diet‐MEF2 interactions shape lipid droplet diversification in muscle to influence Drosophila lifespan
Source: Aging Cell. 2020 Jun 14;19(7):e13172. doi: 10.1111/acel.13172 (PMC7433001; doi:10.1111/acel.13172)
Supplement: Supplementary file 2 — Supplementary Material [file ACEL-19-e13172-s002.pdf]

Supplementary Information for:

Diet-MEF2 Interactions Shape Lipid Droplet Diversification in Muscle to Influence Drosophila Lifespan

Xiao Zhao, Xiaotong Li, Xiangyu Shi, and Jason Karpac

Jason Karpac
Email: karpac@tamu.edu

This file includes:

Supplementary Text – Materials and Methods
Figures S1 to S6
Tables S1 to S2
SI References

Supplementary Information Text

Materials and Methods

Drosophila Stocks and Culture

The following strains were obtained from Bloomington Drosophila Stock Center: w^{1118}; UAS-MEF2$^{RNAi\ TRiP}$ (TRiP; Bloomington 38247); UAS-CycE.R (Bloomington 30725); UAS-CycE.L (Bloomington 4781); UAS-CycA (Bloomington 6633); UAS-Luciferase^{RNAi} (Bloomington 31603); Act88FGal4 (Bloomington 38461); UAS-Pink1$^{RNAi\ TRiP}$ (Bloomington 31262), and UAS-ND-24$^{RNAi\ TRiP}$ (Bloomington 51855). The following strains were obtained from Vienna Drosophila RNAi Center: UAS-CycE^{RNAi} (GD47941) and UAS-MEF2^{RNAi} (GD15549). The UAS-LD-GFP strain was kindly provided by M. Welte. Act88FGeneSwitch transgenic flies were generated and characterized in our lab (Mlih et al. 2018).

The high calorie (HC) diet (cornmeal-based) was made with the following protocol: 10g Agar/ 110g Malt Extract/ 27.5g Dry yeast/ 52g Cornmeal/ 3.125ml propionic acid/ 2g Methyl 4-Hydroxybenzoate/ 1.0L water. The standard SY diet was made with the following protocol (as previously described in (Skorupa et al. 2008)): 10g agar/100g sucrose/ 100g yeast/ 3.125ml propionic acid/ 2g Methyl 4-Hydroxybenzoate/ 1.0L water. The high sugar/low yeast diet was made with the following protocol: 10g agar/400g sucrose/ 25g yeast/ 3.125ml propionic acid/ 2g Methyl 4-Hydroxybenzoate/ 1.0L water. The high yeast/low sugar diet was made with the following protocol: 10g agar/ 25g sucrose/ 400g yeast/ 3.125ml propionic acid/ 2g Methyl 4-Hydroxybenzoate/ 1.0L water. Ingredients were combined, heated to 102°C for 1h, and cooled to 70°C before pouring. For RU486 food, RU486 (Sigma, M8046; dissolved in ethanol) or ethanol (-RU486 control) was mixed with food, resulting in a 100µM concentration of RU486 in the food, unless otherwise indicated.

In order to standardize metabolic results, fifty virgins were crossed to 10 males and kept in bottles for 2-3 days to lay enough eggs. Wet folded filters (GE healthcare, CAT No.10311843) were inserted in bottles after parental flies removed. Progeny of crosses was collected for 3–4 d after initial eclosion. Collected progeny were then transferred to new bottles to allow them mate for 2 d. All these flies were reared on high calorie diet (cornmeal-based) at 25°C and 65% humidity on a 12 hr light/dark cycle, unless otherwise indicated. Around 20 female flies were then separated into each vial with diet for 10 days at 25 °C and 65% humidity on a 12 hr light/dark cycle.

The UAS-MEF2^{RNAi} (GD47941), UAS-CycE^{RNAi}, UAS-CycE R, Act88FGal4, and Act88FGS transgenic lines were backcrossed 10x into the w^{1118} background that was used as a control strain, with continued backcrossing every 6-8 months to maintain isogeneity.

Metabolite measurements

To prepare fly homogenates for metabolic measurements, five females (without head) or ten thoraces were homogenized in 200 µl of PBST (PBS, 0.1% Tween 20) and heated at 70°C for 5

min to inactivate endogenous enzymes. Samples were centrifuged at 4000 rpm for 3 min at 4 °C to clear homogenate. To prepare hemolymph for metabolic measurements, the thoraces of 40 flies were carefully pierced with a tungsten needle and then placed in a perforated 0.5 ml Eppendorf tube within a 1.5 ml Eppendorf tube. Then, the pierced flies were centrifuged at 4000 rpm for 3 min at 4 °C. The supernatant was carefully collected to avoid debris. Collected hemolymph was centrifuged again at 4000 rpm for 3 min to precipitate debris. The collected hemolymph was used measure metabolites immediately.

Ten microliters of cleared extract were used to measure triglycerides (TAGs, StanBio Liquicolor Triglycerides Kit) or glucose (Glucose Assay Kit, GAGO-20) or trehalose (Megazyme) or protein concentrations (Bio-Rad Protein Assay) according to the manufacturer instructions. TAGs or glucose or trehalose levels were normalized to weight (for whole animal, measured using MT XS64 scale) or protein level (for thorax and hemolymph). Note: The kit measures glycerol cleaved from TAG and diacylglycerol (DAG), as well as minimal amounts of free glycerol; the majority of neutral lipids extracted from whole flies are TAG(Carvalho et al. 2012; Palm et al. 2012).

Cholesterol measurements

Total and free cholesterol were measured using the Amplex Red Cholesterol Assay Kit (Invitrogen) according to the manufacturer's instructions. Briefly, thoraces from 10 female flies were homogenized in 200 μL PBST (PBS, 0.1% Tween 20), then heat treated for 5 min at 70 ℃ to inactivate endogenous enzymes. After centrifuging for 3 min at 4,000 rpm, 50 μL of supernatant was added to an equal volume of working solution with or without cholesterol esterase. After 30 min incubation at 37 °C, the fluorescence intensity (λex = 530/λem = 590 nm) was measured on a CLARIOstar microplate reader (BMG LABTECH). Total cholesterol and cholesterol ester(s) (total subtracted of free cholesterol values) were determined by normalization to protein levels by using the BCA Protein Assay Kit (Pierce).

Lactate measurements

Lactate concentrations were measured using the Lactate Assay Kit (Sigma-Aldrich) according to the manufacturer's instructions. In brief, thoraces from 10 female flies were homogenized in 200 μL PBST (PBS, 0.1% Tween 20), then heat treated for 5 min at 70 ℃ to inactivate endogenous enzymes. Four volumes of the lactate assay buffer were added to the sample, and centrifuged at 13,000g for 10 min to remove insoluble material. The supernatant was deproteinized with 10 kDa MWCO spin filter (Life Technologies) to remove lactate dehydrogenase. Samples were brought to 50 μL per well with lactate assay buffer, and incubated for 30 min at room temperature. Absorbance (570 nm [A570]) was obtained using an Epoch Microplate Spectrophotometer (BioTek Instruments). Lactate levels were normalized to total protein levels using the BCA Protein Assay Kit (Pierce).

Oil Red O staining

Intact intestines (midguts) and carcasses (with all of the eggs and intact intestines removed) were dissected in PBS and fixed in 4% paraformaldehyde for 20 min, then washed twice with PBS, incubated for 20 min in fresh Oil Red O solution (6 ml of 0.1% Oil Red O in isopropanol and 4 ml distilled water, and passed through a 0.45 µm syringe), followed by rinsing with distilled water.

Immunostaining and microscopy

For muscle immunostaining, longitudinal thorax muscle segments were dissected in PBS and fixed with 4% paraformaldehyde for 20 min at room temperature, washed 3 times with PBS containing 0.2% Triton X-100 (PBST) and then blocked in blocking buffer (5% BSA in PBST) for 1 h. For immunohistochemistry, the primary antibody anti-Ubiquitin from Enzo (BML-PW8805-0500, 1:500) were applied overnight at 4°C. Alexa Flour-conjugated secondary (Jackson Immunoresearch, 1:500) antibodies were incubated for 2 h at room temperature. Hoechst (DAPI; 1:500) was used to stain DNA. Alexa Fluor 555 Phalloidin (Thermo Fisher Scientific, 1:500) was used to stain F-actin filaments.

To visualize LD-GFP tagged lipid droplets in muscle, longitudinal thorax muscle segments were dissected in PBS and fixed with 4% paraformaldehyde for 20 min at room temperature, washed 3 times with PBST for 10 min each, and then stained with DAPI (1:500) and Alexa Fluor 555 Phalloidin (Thermo Fisher Scientific, 1:500).

Confocal images were collected using a Nikon Eclipse Ti confocal system and processed using the Nikon software and Adobe Photoshop.

Nile Red staining

Longitudinal thorax muscle segments were dissected in PBS and fixed with 4% paraformaldehyde for 10 min at room temperature, washed 3 times with PBS for 10 min each, and then incubated with fresh Nile Red solution (2µl of 0.004% Nile Red Solution in 500 µl PBS) for 2 h at room temperature, followed by rinsing with PBS and then staining with DAPI (1:500). Identical protocols were used to visualize Nile Red and LD-GFP positive lipid droplets in muscle segments.

Confocal images were collected using a Nikon Eclipse Ti confocal system (utilizing a single focal plane) and processed using the Nikon software and Adobe Photoshop.

***Ex vivo* CholEsteryl BODIPY staining**

CholEsteryl (CE) BODIPY™ 542/563 C11 (Thermo Fisher Scientific) was used to trace cholesteryl esters in thorax muscle. Briefly, longitudinal thoracic muscle segments were isolated/dissected in PBS and then incubated/stained with CholEsteryl BODIPY (2 µM in PBST) for 1 h at room temperature (Fig. 2E) and imaged live (unfixed). In order to monitor co-staining of CE BODIPY with

LD-GFP+ lipid droplets (Fig. 2F), muscle segments had to be fixed minimally in 4% PFA (for 5 min.), before incubation with CE BODIPY.

If needed, DAPI (1:500) and Alexa Fluor 488 Phalloidin (Thermo Fisher Scientific, 1:500) were added to stain DNA and F-actin filaments, respectively. Confocal images were collected immediately after CE BODIPY incubation/staining using a Nikon Eclipse Ti confocal system and processed using the Nikon software and Adobe Photoshop.

Food (Calorie) intake / Feeding behavior analysis

For measuring food intake/calorie intake (Fig. 1A and previously described in (Skorupa et al. 2008)); feeding assays on blue dye-labeled food were done as follows: 30 flies were transferred from various diets (high calorie or HS-LY or HY-LS) to vials filled with identical dietary media containing 0.5% brilliant blue (dye). Feeding was interrupted after 1h and 5 flies each were transferred to 50 µl 1 x PBS containing 0.1% Triton X-100 (PBST) and homogenized immediately. Blue dye consumption was quantified by measuring absorbance of the supernatant at 630 nm (A630). Various amounts of dye-containing food were weighed, homogenized in PBST, and measured (A630) in order to create a standard curve used to quantify blue dye food consumption.

For measuring food intake/feeding behavior (Fig. S5); the CAFE assay (Deshpande et al. 2014) was done as follows: Briefly, a single fly was transferred to vials filled with 5 ml of 1.5% agar that maintains internal humidity and serves as a water source. Flies were fed with 5% sucrose solution maintained in 5 ul capillaries (VWR, #53432-706). After twelve hours habituation, the old capillaries were replaced with a new one at the start of the assay. The amount of liquid food consumed was recorded after 24 hr and corrected on the basis of the evaporation (typically < 10% of ingested volumes) observed the identical vials without flies. 5 flies were weighed in order to normalize samples.

RNA-Seq analysis

Intact fly thoraces (8) were dissected in PBS. Total RNA was extracted using Trizol reagent and used as template to generate sample libraries for RNA sequencing (using the TruSeq Stranded Total RNA Library Prep Kit). Sample libraries were sequenced using the Illumina NextSeq 500. Sequence cluster identification, quality pre-filtering, base calling and uncertainty assessment were done in real time using Illumina's HCS and RTA software with default parameter settings. Between 8 and 10 million (2X150) base pair reads were generated per library and mapped to the Drosophila genome (Release 6). Expression was recorded as TPM (transcripts per kbp per million reads) followed by Log_2 transformation. Gene Ontology clustering analysis was performed using FlyMine. FASTQ data files representing unique libraries were deposited in the NCBI Gene Expression Omnibus database (GSE147676).

de novo lipid synthesis analysis

After ten days of feeding on indicated diets, files were transferred to a blue dye (0.5% brilliant blue)-labeled diet with 2µCi of 14C-labeled glucose for 2h. Files with blue dye were then transferred to fresh diet without 14C-labeled glucose for 5h, thoraces (5) were dissected in PBS and total lipid was extracted. For extraction of total lipid, 10 thoraces or 10 carcasses for each sample were homogenized in 2 ml Folch reagent (CHCl3: MeOH 1:1 v/v). Add 0.4 ml cold 0.1 M KCl, vortex 1 min and then spin at 3000 rpm at 4°C for 5 min, then transferred lower phase to new glass tube and dried down. The dried lipid was re-suspended in scintillation fluid, and counted (CPM). Zero-hour samples indicate the rate of incorporation of glucose into fatty acids and 24-hour samples indicate the breakdown of the labeled fatty acids.

Quantification of LD-GFP positive lipid droplets and poly-Ubiquitin

For the quantification of lipid droplet size/area and numbers, about 6-18 randomly selected confocal images from different samples were processed by using ImageJ software (version 1.52, NIH, https://imagej.nih.gov/ij/). Lipid droplets were marked as a region of interest (ROI) by free hand selection tool, then assessed their size and numbers. An identical protocol was used to quantify poly-Ubiquitin.

Climbing assay

Twenty flies were placed into the empty vials, tapped to the bottom, and then given 30 sec. to climb a distance of 6 cm. Flies that successfully climbed 6 cm or beyond in 30 sec were counted. At least 100 total flies (5 cohorts) were used for each genotype tested.

Lifespan analysis

Fifty virgins were crossed to 10 males in bottles for all lifespan experiments unless otherwise indicated. The mated parental files were kept in bottles for 2-3 days to lay enough eggs. Wet folded filter (GE healthcare, CAT No.10311843) were inserted in bottles after parental flies removed. Progeny of crosses was collected for 3–4 d after initial eclosion. Progeny were then transferred to new bottles to mate for 2 d. Around 100 flies were then separated according to sex and genotype into each cage and aged at 25 °C with constant humidity (approximately 65%).

For all independent populations, plastic cages (175-ml volume, 5-cm diameter from Greiner Bio-One) were used for lifespan experiments. Food, changed every 2–3 d, was provided in vials inserted into a foam plug (4.9 cm in diameter, 3-cm thick from Greiner Bio-One). RU486 or ethanol was mixed with food, resulting in a 100 µM concentration of RU486 in the food (unless otherwise indicated for dose response experiments). Dead flies were counted every 2–3 d. lifespan data were analyzed using Prism statistical software.

All independent lifespan analyses (and statistics) for independent trials are included in the Table S1, and raw data for all lifespan analysis (unique populations) is included in Table S3.

Mortality estimation

Mortality rate was calculated as described (Mair et al. 2003). N0 is the number of individuals in the initial cohort, as well as Nx, the number alive at the start of each day. The probability of surviving from age x to age x + 1, given the individual is alive at the start of age x, is Px = Nx + 1/Nx. The age-specific rate of mortality is estimated as μx = −ln(Px). The mortality rate is plotted as ln(μx).

Analysis of gene expression

Total RNA from intact fly thorax (8) were extracted using Trizol and complementary DNA synthesized using Superscript Ⅲ (Invitrogen). Real-time PCR was performed using SYBR Green, the Applied Biosystems StepOnePlus Real-Time PCR systems, and the primers pairs described in the extended experimental procedures (Table S2). Results are average ± standard error of at least three independent samples, and quantification of gene expression levels calculated using the ΔCt method and normalized to actin5C expression levels.

Transmission electron microscopy (TEM)

Whole thoraxes were fixed in Trump's Fixative buffer (1.16g $NaH_2PO_4 \cdot H_2O$, 0.27g NaOH, 10ml 37-38% formaldehyde, 2ml 50% glutaraldehyde and 88 ml water). Samples were left at room temperature for about an hour, then stored at 4°C.. Fixed samples were washed in Trump's buffer (1.16g of $NaH_2PO_4 \cdot H_2O$, 0.27g NaOH, add water to 100ml, adjust pH to 7.2-7.3) X3, followed by a 1 min microwave @250W each time. Samples were then post-fixed in 1% Osmium tetroxide. Following dehydration with an acetone series, samples were embedded in Modified Quetol-ERL 4221 Resin (Quetol651 1.93g, ERL4221 1.64g, NSA 6.43g, DER736 1.0g, BDMA 0.25 ml). Ultrathin sections (85 nm) were cut at various levels of each block and stained with 2% uranyl acetate and lead citrate. Images were captured with a FEI TECNAI G2 F20 FE-TEM transmission electron microscope.

Statistical analysis

For all experiments, the data is represented as mean ± SE. All p-values were calculated using the Student's t-test excluding the lifespan analysis. Lifespan data was analyzed using Prism statistical software, and Log-rank and Wilcoxin Chi Square analysis were used to calculate p-values.

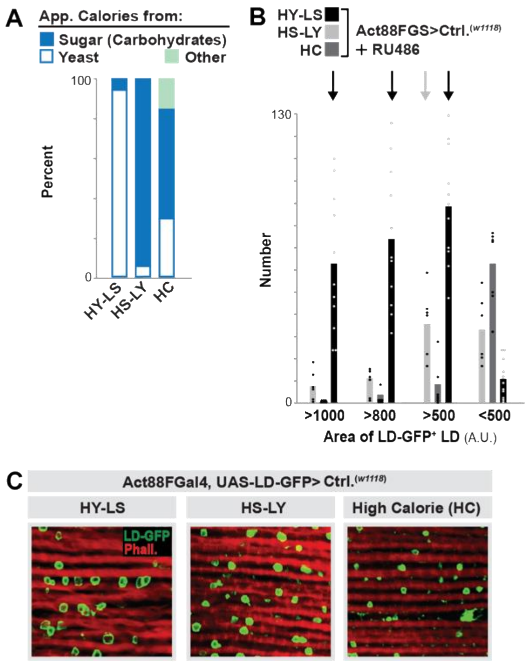

Figure S1: Diet-dependent Control of Intramuscular Lipid Droplet Accumulation.

(A) Approximate (App.) percentages of calorie sources in various diets.

(B) Quantification of lipid droplet (LD) size/area (LD-GFP-positive lipid droplets) from indicated diets (plotted as number of individual droplets of various sizes [A.U., arbitrary units >1000, >800, >500, or <500]). Genotype Act88FGS, UAS-LD-GFP>w^{1118} (+, control); all flies fed RU486 (Representative images presented in Figure 1). n=480-1119 LDs from 6-11 independent dissected muscle segments.

(C) Fluorescent imaging and staining to detect LD in dissected dorsal longitudinal thoracic muscle from indicated diets; LD (LD-GFP, green) and F-actin filaments (Phalloidin, Red). Genotype Act88FGal4, UAS-LD-GFP>w^{1118} (+, control).

Bars represent mean. All experiments represent female flies.

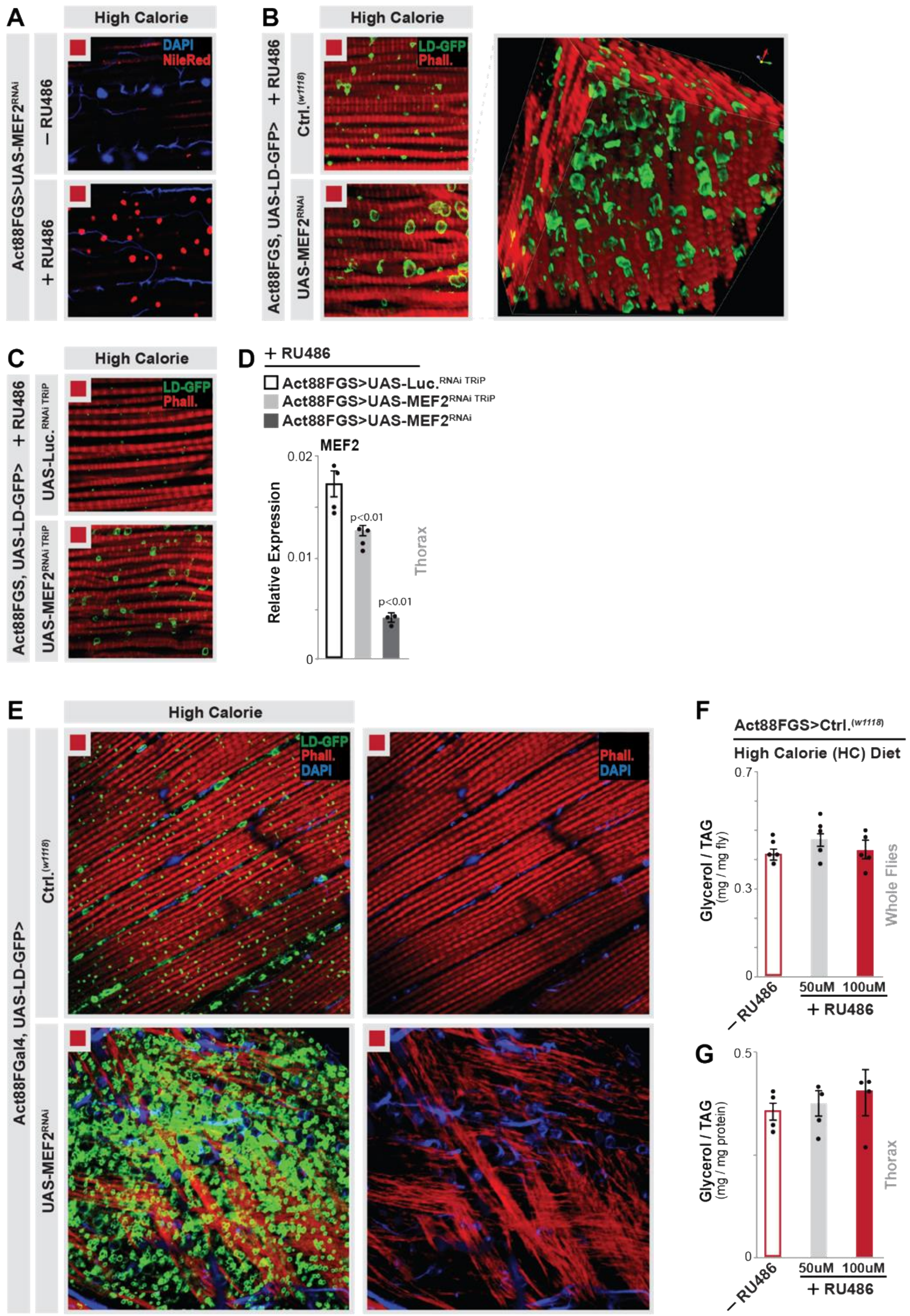

Figure S2: MEF2 Controls Intramuscular Lipid Droplet Accumulation in Response to High Calorie Diet.

(A) Staining of neutral lipids (Nile Red, Red) in dissected dorsal longitudinal thoracic muscle (nuclei [DAPI, blue]); Act88FGS>UAS-MEF2 RNAi flies, ±RU486 (high calorie diet).

(B) Additional examples of fluorescent imaging and staining to detect lipid droplets (LD) in dissected dorsal longitudinal (DL) thoracic muscle (high calorie diet); LD (LD-GFP, green) and F-actin filaments (Phalloidin, red). Genotype Act88FGS, UAS-LD-GFP>UAS-MEF2 RNAi; all flies fed

RU486. Right panel represents 3D reconstruction (confocal Z-stack) of dissected DL segment highlighting intermyofibrillar LDs.

(C) Fluorescent imaging and staining to detect LD in dissected dorsal longitudinal thoracic muscle (high calorie diet); LD (LD-GFP, green) and F-actin filaments (Phalloidin, Red). Genotypes Act88FGS, UAS-LD-GFP>Luciferase (Luc) RNAi (control) or Act88FGS, UAS-LD-GFP>UAS-MEF2 RNAi^{TRiP}; all flies fed RU486.

(D) UAS-MEF2 RNAi efficiency. Changes in *MEF2* expression (measured by qRT-PCR, plotted as relative expression) from dissected thoraces/muscle upon depletion of MEF2 (MEF2 RNAi [VDRC: GD15549] and MEF2 RNAi^{TRiP} [TRiP: Bloomington 38247]) using the Act88FGS driver (compared to Luciferase RNAi controls); all flies fed RU486. n=4 samples.

(E) Fluorescent imaging and staining to detect LD in dissected dorsal longitudinal thoracic muscle from indicated diets; LD (LD-GFP, green), F-actin filaments (Phalloidin, Red), and nuclei (DAPI, blue). Genotypes Act88FGal4, UAS-LD-GFP>w^{1118} (+, control) and Act88FGal4, UAS-LD-GFP>UAS-MEF2 RNAi.

(F) Triglyceride (TAG) content from whole flies. Act88FGS>w^{1118} (+, control) flies, indicated doses of RU486 or mock (- RU486) control; (high calorie diet). n=5 samples.

(G) TAG content from dissected thoraces/muscle. Act88FGS>w^{1118} (+, control) flies, indicated doses of RU486 or mock (- RU486) control; (high calorie diet). n=4 samples.

Bars represent mean±SE. All experiments represent female flies.

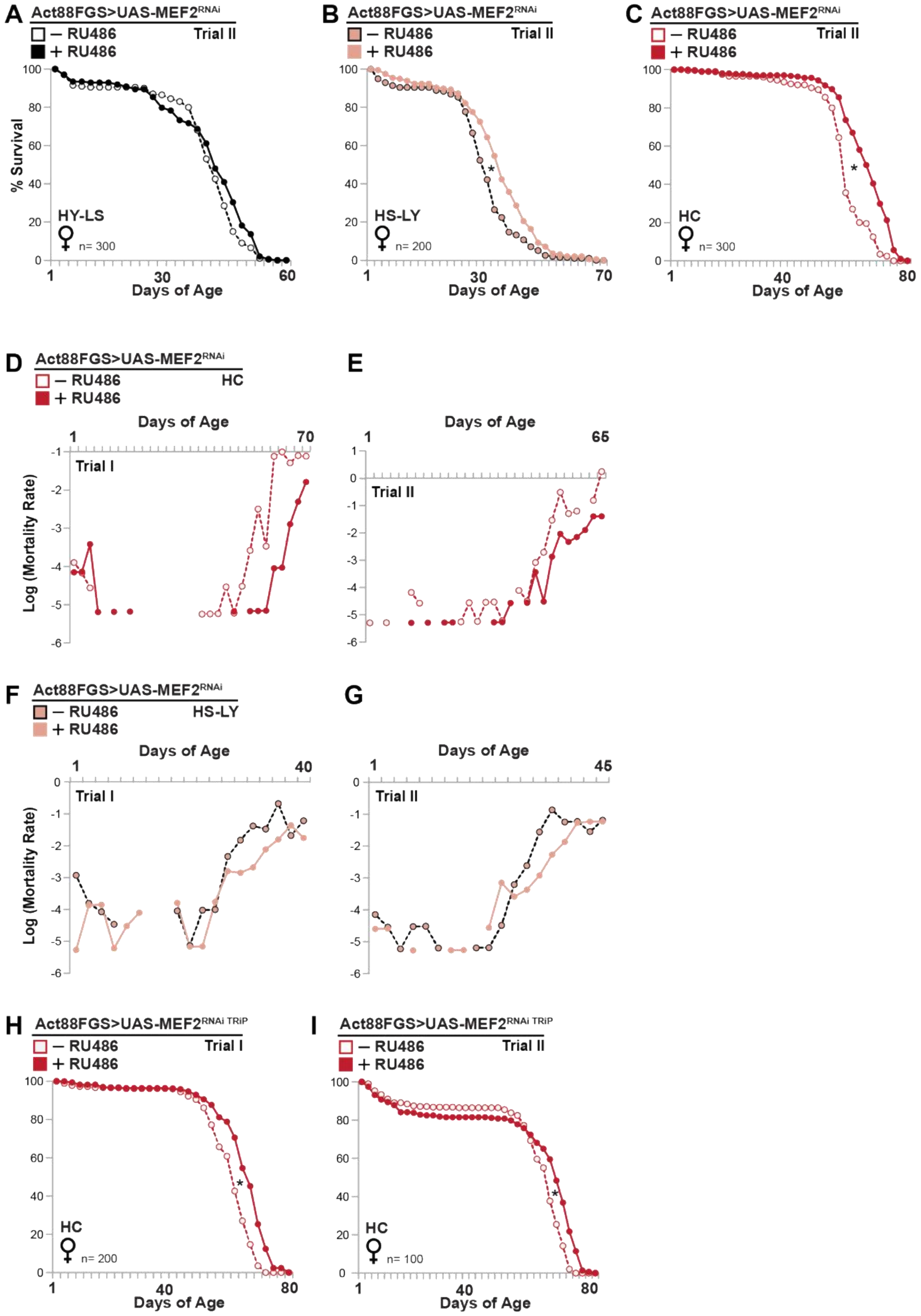

Figure S3: Intramuscular Lipid Droplet Diversification Correlates With Enhanced lifespan and Decreases in Age-dependent Mortality.

(A-C) Lifespan curves (lifespan, independent trial II); Act88FGS>UAS-MEF2 RNAi female flies (±RU486) fed a (A) HY-LS diet, (B) HS-LY diet, or (C) high calorie (HC) diet. n=200-300 flies; *p value<0.01 (mean lifespan).

(D-G) Mortality plots corresponding to lifespan analysis found in Fig. 2B-C and Fig. S3B-C. Act88FGS>UAS-MEF2 RNAi female flies (±RU486) fed a (D) HC diet, trial I, (E) HC diet, trial II, (F) HS-LY diet, trial I, and (G) HS-LY diet, trial II.

(H-I) Lifespan curves (lifespan, independent trials I and II); Act88FGS>UAS-MEF2 RNAi^{TRiP} (H) trial I and (I) trial II; female flies (±RU486) fed a HC diet. n=100-200 flies.

All experiments represent female flies. * p value < 0.001 (mean lifespan).

A

	Act88FGS>			
	Ctrl. (w1118)		UAS-MEF2 RNAi	
RU486	- (EtOH)	+	- (EtOH)	+
CG14757	1.267931065	1.009776105	1.305728373	0.598172477
CG34423	1.241914204	1.090514788	1.054144578	0.700449239
Rim2	1.112232953	1.027887043	1.288293944	0.678959525
CG15412	1.094262467	1.009258718	1.366337218	0.662701707
Nc73EF	1.079423517	1.046190961	1.310820246	0.67554459
CG8613	1.073976617	1.067773744	1.226042722	0.711246812
clumsy	1.064609309	1.101599603	1.189937038	0.716575553
CG8475	1.063155602	1.11277626	1.162993834	0.726804996
Las	1.059970952	1.028469158	1.347792794	0.680599512
Npl4	1.058026129	1.014114952	1.415201187	0.658564379
Cpr73D	1.046843425	1.083091092	1.270390235	0.694250402
CR44116	1.043859252	1.122378317	1.171436014	0.728618536
MagR	1.021722051	1.090166454	1.271039747	0.706342418
Ucp4A	1.020451077	1.203624934	1.176035608	0.692302921
Fhos	1.011159438	1.071268666	1.352698174	0.682466053
Alk	1.004208871	1.130853531	1.172033736	0.75132783
CG1674	1.004017395	1.177109628	1.129679824	0.749008038
rdgA	1.00381778	1.104969215	1.224563416	0.736230249
SdhA	1.003757982	1.133232448	1.238446142	0.709863527
CG8768	1.003070187	1.090449404	1.267852541	0.721098201
D2hgdh	0.996781227	1.150438945	1.168154984	0.746510823
AQP	0.991217946	1.250769151	1.112530662	0.725006154
bt	0.984467734	1.133703958	1.238092223	0.723678807
CG18208	0.983281033	1.13920359	1.20611137	0.740173584
Frq2	0.981934128	1.155838159	1.192513212	0.73885194
CG7083	0.980294391	1.187053075	1.146743624	0.749388477
CG34180	0.978933856	1.15153457	1.207338749	0.734751585
ND-51	0.978901986	1.163477172	1.236684758	0.709976405
CG6329	0.977839656	1.151168678	1.159006855	0.766491574
PheRS-m	0.977708281	1.073223854	1.356269219	0.70267497
ND-24	0.977140205	1.140612645	1.269236606	0.706907104
Strn-Mlck	0.976530691	1.091808239	1.310763871	0.715555416
tobi	0.975025531	1.379243511	1.097135254	0.67777086
cpo	0.967728687	1.171429312	1.169348236	0.754373516
Pcmt	0.966014961	1.147784204	1.232297945	0.731880506
trbd	0.966005287	1.071013674	1.355231635	0.713201011
CG8520	0.96450723	1.12760165	1.283540334	0.716356638
sals	0.964173121	1.203340038	1.161901591	0.741800747
CG42329	0.963767078	1.242608945	1.167957744	0.714934586
Acon	0.960645564	1.322013233	1.138352523	0.691710299
CG17816	0.957920934	1.150728494	1.184016016	0.766196019
Pkc98E	0.956767564	1.164217112	1.180492043	0.760495264
ND-20	0.955864383	1.164536966	1.239362718	0.72485646
CG15237	0.954257124	1.189253328	1.203863082	0.731952868
CG7766	0.953396405	1.117485914	1.307681062	0.717765531
QIL1	0.951967362	1.142997844	1.206139672	0.761964832
CR44961	0.949343343	1.127698866	1.271292583	0.73474736
CG5246	0.948055561	1.288640974	1.129456576	0.724710803
CG4733	0.948022677	1.12818465	1.297528392	0.720583341
dnc	0.945772466	1.194720401	1.153202877	0.767434531
ND-23	0.943927642	1.123626685	1.283516037	0.734578058
Glycogenin	0.941273613	1.137523108	1.246335751	0.749357114
Tet	0.940496339	1.164544977	1.189803797	0.76738141
Unc-89	0.940313058	1.287308864	1.116328767	0.740035676
Pfk	0.940102721	1.148696507	1.234796754	0.749935497
Pink1	0.938638966	1.14501216	1.265853215	0.735034921
Pask	0.934601171	1.270514508	1.12893511	0.74597637
Mef2	0.93400656	1.168688662	1.189894827	0.769914797
slgA	0.932676112	1.114922903	1.31439329	0.731642586
mRF1	0.932565178	1.119760132	1.287132048	0.743999597
Eno	0.930749913	1.175630483	1.245162826	0.733955976
IscU	0.930018035	1.164251093	1.224289387	0.754358678
tau	0.928680776	1.185573721	1.179966424	0.769724536
Neto	0.926991508	1.201509776	1.163940111	0.771376316
SdhB	0.924776603	1.241887224	1.212001702	0.718418946

B

	Act88FGS>			
	Ctrl. (w1118)		UAS-MEF2 RNAi	
RU486	- (EtOH)	+	- (EtOH)	+
dhd	0.636943606	0.625521442	0.773606881	3.24441569
osk	0.646365344	0.655618772	0.909111121	2.595694558
CG17018	0.66117195	0.692909771	0.776334502	2.811641895
yl	0.664144294	0.675386903	0.92534876	2.409236713
spd-2	0.67024636	0.903724748	0.939675219	1.756918381
wisp	0.673679501	0.669165459	0.94319599	2.351858593
Pxt	0.675248298	0.689267587	0.809018527	2.655768272
CycA	0.679565178	0.688861098	0.910326049	2.346606412
CycB	0.688056566	1.137394906	0.686152752	1.862274806
Hsp27	0.690671291	0.812536181	1.028881428	1.731891017
otu	0.696732839	0.708383337	0.87905528	2.304884648
alphaTub67C	0.707687054	0.741090283	0.873778857	2.182157526
polo	0.715156273	0.779806527	0.818527083	2.190680932
snRNA:7SK	0.717306325	0.746452439	1.088917299	1.715134968
CG14814	0.723522917	0.849899191	0.954305586	1.704091173
rpk	0.724628535	0.732426696	1.082591143	1.740427485
PCNA	0.730040347	0.938447816	0.86047791	1.696302429
BigH1	0.730313577	0.789991793	0.806094003	2.150217317
mtrm	0.731568159	0.723835772	0.809893657	2.331724122
tum	0.732155298	0.797801159	1.044353477	1.639285551
gwl	0.735569724	0.789005282	0.938130489	1.836677871
RpS5b	0.73914805	0.756517633	0.927610903	1.927896277
nos	0.746912631	0.761123678	0.841956866	2.08922455
stg	0.748035975	0.928155797	0.917428044	1.569945666
Pen	0.751821946	0.912031063	0.725951243	2.0089444
gnu	0.757419193	0.765807276	0.840108508	2.05214868
CycB3	0.758822386	0.85846	0.858699723	1.787715801
Cyp6a19	0.761313455	0.757504977	0.917503655	1.889919043
Cdk1	0.764488808	0.819845022	0.918339611	1.737375877
CycE	0.770233897	0.868979911	0.865699713	1.725839333
CG6927	0.7712479	0.850190684	0.968434025	1.574779034
fs(1)Ya	0.773284349	0.781321945	0.837106579	1.977197267
Mcm5	0.776257913	0.95148337	0.920069713	1.471539835
CR44155	0.782774029	0.789716631	0.993834597	1.627714366
asp	0.786887755	0.846223232	0.851632074	1.763397656
swa	0.788958585	0.862823569	0.802253718	1.831099969
CG18190	0.790779789	0.912296363	0.727751239	1.90469544
CG7208	0.791012895	0.84770165	0.963740887	1.547437524
CG14545	0.791732754	0.826883489	0.875798999	1.744104982
retn	0.79237439	0.848323453	0.929773854	1.60003973
APC7	0.793525626	0.871629592	0.979544607	1.475988111
CG4570	0.794163875	0.918652777	0.898121309	1.526171992
CBP	0.794673426	0.778787525	0.891386305	1.8127018
feo	0.811149303	0.829380766	0.897583067	1.656039115
vas	0.815114739	0.967243689	0.86790115	1.46142013
zpg	0.815650361	0.826191422	0.853340094	1.738974183
AGO3	0.818285026	0.798508699	0.956548716	1.599958255
dah	0.81876682	0.80662971	0.851659433	1.777868316
CG14036	0.824950037	0.831352451	0.870099525	1.675784777
pim	0.828860604	0.942668979	0.892932667	1.433311464
CG11674	0.834453997	0.844452033	0.873035467	1.62551405
krimp	0.839054488	0.875495751	0.897389321	1.516962677
borr	0.840916173	0.821508795	0.858416787	1.686307905
CG5194	0.84100384	0.876706859	0.885617696	1.53144474
CG4476	0.84598293	0.813445479	0.947179025	1.534185515
Orc2	0.851373656	0.889524819	0.884475227	1.492918326
WRNexo	0.855273031	0.841702028	0.909664573	1.527058122
sub	0.856967025	0.861996458	0.947982077	1.428006819
Chd3	0.865602015	0.90323124	0.91866561	1.392275985
sip2	0.867489886	0.813321749	0.894255868	1.584934778
CG13690	0.8784686	0.915526462	0.896230758	1.387340329
bora	0.880618671	0.885057477	0.90381358	1.419586213
gammaTub37C	0.895561754	0.873548813	0.914675355	1.39749514
Klp61F	0.911360362	0.884984238	0.879933713	1.409043405
bcd	0.914658738	0.890737559	0.884061408	1.388381168

Figure S4: Muscle-specific and MEF2-dependent Changes in Gene Expression.

(A-B) Gene expression levels (Log_2 transformed values) from RNA-seq. transcriptomics analysis (dissected thoraces/muscle) from Act88FGS>*w*^{1118} (+, control) and Act88FGS>UAS-MEF2 RNAi flies (±RU486, high calorie diet). (A) Table includes genes down-regulated in MEF2 RNAi flies (fold change <=0.65 MEF2 RNAi +RU486/-RU486 without down-regulation in controls) with gene names and Log_2 transformed values (represents data from heat map presented in Fig. 4A. (B) Table includes genes up-regulated in MEF2 RNAi flies (fold change >=1.5 MEF2 RNAi +RU486/-RU486

without up-regulation in controls) with gene names and Log_2 transformed values (represents data from heat map presented in Fig. 5A.

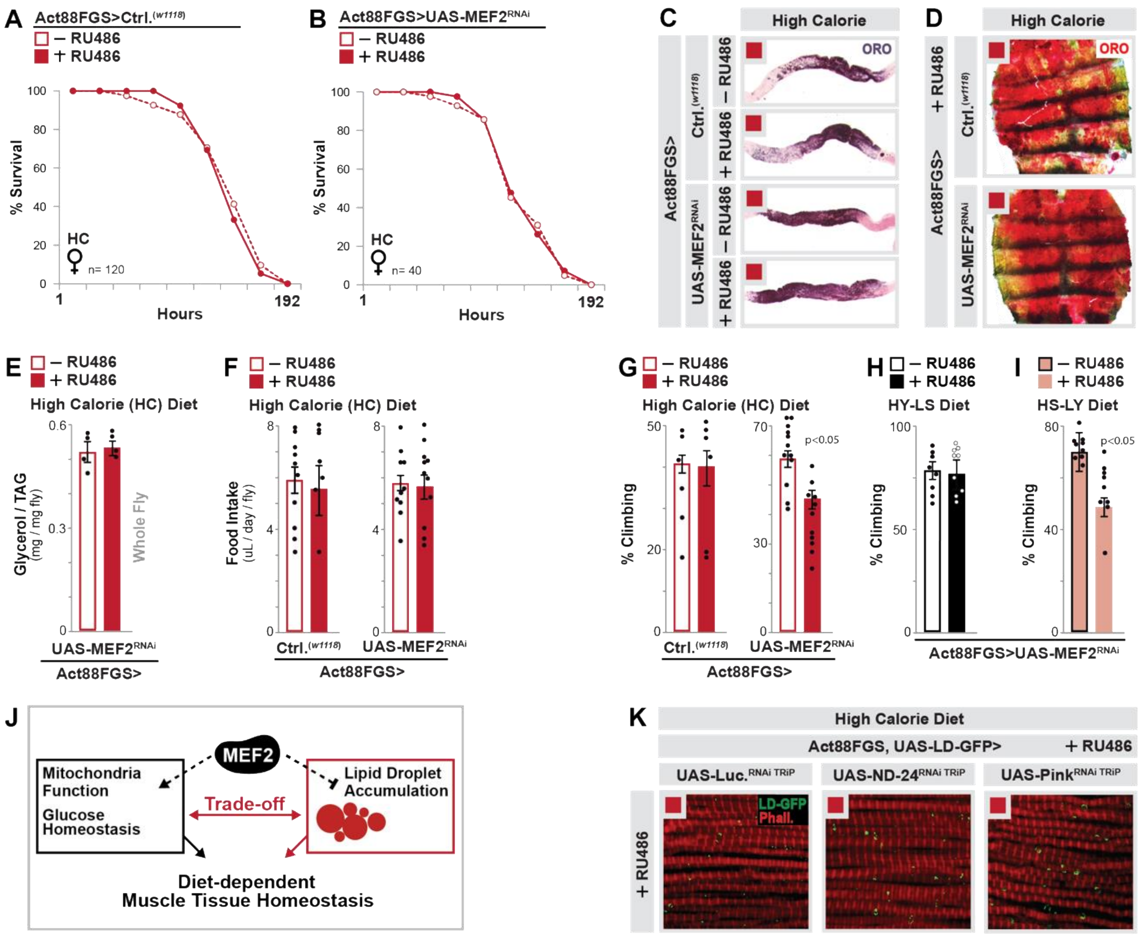

Figure S5: Systemic and Autonomous Effects on Physiology in Response to Muscle-specific Diet-MEF2 Interactions.

(A-B) Starvation sensitivity of female flies. Genotypes (A) Act88FGS>*w*^{1118} (+, control) and (B) Act88FGS>UAS-MEF2 RNAi; (±RU486) fed a high calorie (HC) diet. n=40-120 flies.

(C) Oil red O (ORO) neutral lipid stain of dissected midguts; genotypes described above (high calorie diet).

(D) ORO stain of dissected fat body/carcass; genotypes described above (high calorie diet).

(E) Triglyceride (TAG) content from whole flies; genotype described above (±RU486, high calorie diet). n=4 samples.

(F) Feeding behavior (food intake analysis; CAFE assay); genotypes described above (high calorie diet). n=8-10 individual flies.

(G-I) Motility (climbing assay); (G) Act88FGS>*w*^{1118} (+, control) and Act88FGS >UAS-MEF2 RNAi (±RU486) fed a HC diet, (H) Act88FGS >UAS-MEF2 RNAi (±RU486) fed a HY-LS diet, and (I) Act88FGS >UAS-MEF2 RNAi (±RU486) fed a HS-LY diet. n=6-12 cohorts of 20 flies.

(J) Model depicting trade-off between MEF2-dependent control of mitochondria function/glucose homeostasis and lipid droplet accumulation, which governs muscle tissue homeostasis.

Bars represent mean±SE. All experiments represent female flies.

(K) Fluorescent imaging and staining to detect LD in dissected dorsal longitudinal thoracic muscle (high calorie diet); LD (LD-GFP, green) and F-actin filaments (Phalloidin, Red). Genotypes Act88FGS, UAS-LD-GFP>Luciferase (Luc) RNAi (control); Act88FGS, UAS-LD-GFP>UAS-ND-24 RNAi^{TRiP}; or Act88FGS, UAS-LD-GFP>UAS-Pink1 RNAi^{TRiP}; all flies fed RU486.

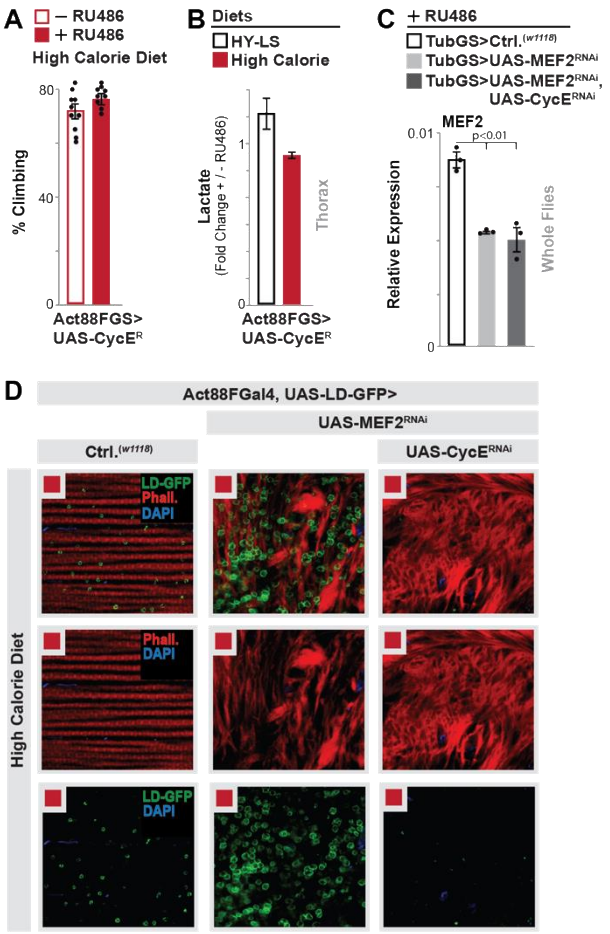

Figure S6: Autonomous Effects on Physiology and Morphology in Response to Muscle-specific Genetic Manipulation of Cyclin E.

(A) Motility (climbing assay); Act88FGS>UAS-CycE R (±RU486) fed a high calorie (HC) diet. n=5 cohorts of 20 flies.

(B) Lactate levels from dissected thoraces/muscle. Genotype Act88FGS>UAS-CycE R (±RU486) fed a HC or HY-LS diet. Plotted as fold change (ratio +RU486/-RU486). n=4 samples.

Bars represent mean±SE. All experiments represent female flies.

(C) UAS-MEF2 RNAi efficiency. Changes in *MEF2* expression (measured by qRT-PCR, plotted as relative expression) from whole flies upon depletion of MEF2 (MEF2 RNAi [VDRC: GD15549]) and CycE (RNAi) using the TubGeneSwitch (GS) driver (compared to w^{1118} [+] controls); all flies fed RU486. n=3 samples.

(D) Fluorescent imaging and staining to detect LD in dissected dorsal longitudinal thoracic muscle from indicated diets; LD (LD-GFP, green), F-actin filaments (Phalloidin, Red), and nuclei (DAPI, blue). Genotypes Act88FGal4, UAS-LD-GFP>w^{1118} (+, control), Act88FGal4, UAS-LD-GFP>UAS-MEF2 RNAi, and Act88FGal4, UAS-LD-GFP>UAS-MEF2 RNAi; UAS-CycE^{RNAi}. Inhibiting CycE

rescues MEF2-dependent changes in LD accumulation/size but not changes in myofibril morphology/organization.

Table S1: Table summarizing parameters and statistics for lifespan analysis.

									Chi Square		p Value	
Trial	Genotype	Food	RU486	Gender	Populations (n)	Flies (n)	Median Survival	Percent Change	Log-rank	Wilcoxon	Log-rank	Wilcoxon
1	w^{1118};; Act88FGS/UAS-MEF2^{RNAiGD}	HC	-	Female	3	300	56	-	-	-	-	-
	w^{1118};; Act88FGS/UAS-MEF2^{RNAiGD}	HC	+ (100 mM)	Female	3	300	64	+ 14%	80.45	74.37	<0.0001	<0.0001
2	w^{1118};; Act88FGS/UAS-MEF2^{RNAiGD}	HC	-	Female	2	200	62	-			-	-
	w^{1118};; Act88FGS/UAS-MEF2^{RNAiGD}	HC	+ (100 mM)	Female	2	200	71	+15%	117.9	109.8	< 0.0001	< 0.0001
1	w^{1118};; Act88FGS/UAS-MEF2^{RNAiHM}	HC	-	Female	2	200	64	-	-	-	-	-
	w^{1118};; Act88FGS/UAS-MEF2^{RNAiHM}	HC	+ (100 mM)	Female	2	200	68	+ 6%	32.52	18.27	< 0.0001	< 0.0001
2	w^{1118};; Act88FGS/UAS-MEF2^{RNAiHM}	HC	-	Female	1	100	64	-	-	-	-	-
	w^{1118};; Act88FGS/UAS-MEF2^{RNAiHM}	HC	+ (100 mM)	Female	1	100	68	+ 6%	17.03	8.224	< 0.0001	0.0041
1	w^{1118};; Act88FGS/UAS-MEF2^{RNAiGD}	HSLY	-	Female	2	200	34	-	-	-	-	-
	w^{1118};; Act88FGS/UAS-MEF2^{RNAiGD}	HSLY	+ (100 mM)	Female	2	200	39	+15%	20.42	27.14	<0.0001	<0.0001
2	w^{1118};; Act88FGS/UAS-MEF2^{RNAiGD}	HSLY	-	Female	2	200	36	-	-	-	-	-
	w^{1118};; Act88FGS/UAS-MEF2^{RNAiGD}	HSLY	+ (100 mM)	Female	2	200	41	'+ 14%	19.85	24.1	<0.0001	<0.0001
1	w^{1118};; Act88FGS/UAS-MEF2^{RNAiGD}	HYLS	-	Female	2	200	41	-	-	-	-	-
	w^{1118};; Act88FGS/UAS-MEF2^{RNAiGD}	HYLS	+ (100 mM)	Female	2	200	43	+ 4%	3.194	2.491	0.0739	0.1145
2	w^{1118};; Act88FGS/UAS-MEF2^{RNAiGD}	HYLS	-	Female	2	200	41	-	-	-	-	-
	w^{1118};; Act88FGS/UAS-MEF2^{RNAiGD}	HYLS	+ (100 mM)	Female	2	200	41	0%	1.135	0.1601	0.2867	0.6891
1	w^{1118};; Act88FGS/+W1118	HC	-	Female	3	300	56	-	-	-	-	-
	w^{1118};; Act88FGS/+W1118	HC	+ (100 mM)	Female	3	300	56	0%	2.881	0.9469	0.0896	0.3305
2	w^{1118};; Act88FGS/+W1118	HC	-	Female	2	200	67	-	-	-	-	-
	w^{1118};; Act88FGS/+W1118	HC	+ (100 mM)	Female	2	200	62	-8%	61.37	52.26	< 0.0001	< 0.0001
1	w^{1118};; Act88FGS/+W1118	HSLY	-	Female	2	200	43	-	-	-	-	-
	w^{1118};; Act88FGS/+W1118	HSLY	+ (100 mM)	Female	2	200	43	0%	1.558	0.05557	0.2119	0.8136
2	w^{1118};; Act88FGS/+W1118	HSLY	-	Female	3	300	47	-	-	-	-	-
	w^{1118};; Act88FGS/+W1118	HSLY	+ (100 mM)	Female	3	300	47	0%	0.1988	0.2772	0.6557	0.2772
1	w^{1118};; Act88FGS/+W1118	HYLS	-	Female	3	300	43	-	-	-	-	-
	w^{1118};; Act88FGS/+W1118	HYLS	+ (100 mM)	Female	3	300	39	- 10%	27.57	22.87	<0.0001	<0.0001
2	w^{1118};; Act88FGS/+W1118	HYLS	-	Female	3	300	43	-	-	-	-	-
	w^{1118};; Act88FGS/+W1118	HYLS	+ (100 mM)	Female	3	300	41	- 5%	9.953	7.117	0.0016	0.0076
1	w^{1118};; Act88FGS/UAS-MEF2^{RNAiGD}	HC	-	Male	2	200	66	-	-	-	-	-
	w^{1118};; Act88FGS/UAS-MEF2^{RNAiGD}	HC	+ (100 mM)	Male	3	300	64	- 3%	1.069	6.753	0.3012	0.0094
1	w^{1118};; Act88FGS/UAS-MEF2^{RNAiHM}	HC	-	Male	3	300	66	-	-	-	-	-
	w^{1118};; Act88FGS/UAS-MEF2^{RNAiHM}	HC	+ (100 mM)	Male	3	300	58	- 13%	49.51	43.72	<0.0001	<0.0001
1	w^{1118};; Act88FGS/+W1118	HC	-	Male	3	300	58	-	-	-	-	-
	w^{1118};; Act88FGS/+W1118	HC	+ (100 mM)	Male	3	300	47	- 24%	165.8	171.8	<0.0001	<0.0001
1	w^{1118};; Act88FGS/+Luciferase^{RNAi}	HC	-	Male	3	300	60	-	-	-	-	-
	w^{1118};; Act88FGS/+Luciferase^{RNAi}	HC	+ (100 mM)	Male	3	300	53	- 13%	79.67	87.07	<0.0001	<0.0001
1	w^{1118};; Act88FGS/UAS-MEF2^{RNAiGD},CycE^{RNAiGD}	HC	-	Female	2	200	44	-	-	-	-	-
	w^{1118};; Act88FGS/UAS-MEF2^{RNAiGD},CycE^{RNAiGD}	HC	+ (100 mM)	Female	2	200	44	0%	0.01034	0.0003244	0.919	0.9856
1	w^{1118};; Act88FGS/UAS-CycE^{R}	HC	-	Female	2	200	50	-	-	-	-	-
	w^{1118};; Act88FGS/UAS-CycE^{R}	HC	+ (100 mM)	Female	2	200	56	+12%	117.9	99.4	<0.0001	<0.0001

Table S2: qRT-PCR Primer Sequences.

Primer	Sequence (5'-3')
MEF2 F	ACAACGAGCCCCACGAGTCC
MEF2 R	GAGTGAGTGTGTAGTCCGTTTC
Cyclin E F	AGCTACCAGGAACCTCAGCA
Cyclin E R	GTGTAGACCGAGGAGGCAAC
SdhA F	CATGTACGACACGGTCAAGG
SdhA R	GACCAGTACGATCAGCCACA
ND-24 F	CTGACAAACTGTGCCTCCAA
ND-24 R	ATGCTGAGTATGGCCTCCAC
ND-51 F	CAGCGGTATGAAGTGGTCCT
ND-51 R	TGTAGAACTCGCCACGAATG
Pink1 F	AGGAACAGCGATTACCGACA
Pink1 R	TCTTAGTGGTCAGCGAAAGGA
Actin5C F	CTCGCCACTTGCGTTTACAGT
Actin5C R	TCCATATCGTCCCAGTTGGTC

SI References:

Carvalho M, Sampaio JL, Palm W, Brankatschk M, Eaton S & Shevchenko A (2012) Effects of diet and development on the Drosophila lipidome. *Mol Syst Biol* 8, 600.

Deshpande SA, Carvalho GB, Amador A, Phillips AM, Hoxha S, Lizotte KJ & Ja WW (2014) Quantifying Drosophila food intake: comparative analysis of current methodology. *Nat Methods* 11, 535–540.

Mair W, Goymer P, Pletcher SD & Partridge L (2003) Demography of Dietary Restriction and Death in *Drosophila*. *Science* 301, 1731–1733.

Mlih M, Khericha M, Birdwell C, West AP & Karpac J (2018) A virus-acquired host cytokine controls systemic aging by antagonizing apoptosis. *Plos Biol* 16, e2005796.

Palm W, Sampaio JL, Brankatschk M, Carvalho M, Mahmoud A, Shevchenko A & Eaton S (2012) Lipoproteins in Drosophila melanogaster—Assembly, Function, and Influence on Tissue Lipid Composition. *Plos Genet* 8, e1002828.

Skorupa DA, Dervisefendic A, Zwiener J & Pletcher SD (2008) Dietary composition specifies consumption, obesity, and lifespan in Drosophila melanogaster. *Aging Cell* 7, 478–490.
